# Climate in Africa sequentially shapes spring passage of Willow Warbler *Phylloscopus trochilus* across the Baltic coast

**DOI:** 10.7717/peerj.12964

**Published:** 2022-02-18

**Authors:** Magdalena Remisiewicz, Les G. Underhill

**Affiliations:** 1 Bird Migration Research Station, Faculty of Biology, University of Gdańsk, Wita Stwosza, Poland; 2 Department of Biological Sciences, University of Cape Town, Rondebosch, Cape Town, South Africa; 3 Biodiversity and Development Institute, Rondebosch, Cape Town, South Africa

**Keywords:** Sequential migration, Climate change, Spring phenology, Migration timing, Annual anomaly, *Phylloscopus trochilus*, Large-scale climate indices, SOI, IOD, NAO

## Abstract

**Background:**

Many migrant birds have been returning to Europe earlier in spring since the 1980s. This has been attributed mostly to an earlier onset of spring in Europe, but we found the timing of Willow Warblers’ passage to be influenced by climate indices for Africa as much as those for Europe. Willow Warblers’ spring passage through northern Europe involves populations from different wintering quarters in Africa. We therefore expected that migration timing in the early, middle and late periods of spring would be influenced sequentially by climate indices operating in different parts of the winter range.

**Methods:**

Using data from daily mistnetting in 1 April–15 May over 1982–2017 at Bukowo (Poland, Baltic Sea coast), we derived an Annual Anomaly (AA, in days) of Willow Warbler spring migration. We decomposed this anomaly into three main periods (1–26 April, 27 April–5 May, 6–15 May); one-third of migrants in each period. We modelled three sequential time series of spring passage using calendar year and 15 large-scale climate indices averaged over the months of Willow Warblers’ life stages in the year preceding spring migration as explanatory variables in multiple regression models. Nine climate variables were selected in the best models. We used these nine explanatory variables and calculated their partial correlations in models for nine overlapping sub-periods of AA. The pattern of relationships between AA in these nine sub-periods of spring and the nine climate variables indicated how spring passage had responded to the climate. We recommend this method for the study of birds’ phenological responses to climate change.

**Results:**

The Southern Oscillation Index and Indian Ocean Dipole in Aug–Oct showed large partial correlations early in the passage, then faded in importance. For the Sahel Precipitation Index (PSAH) and Sahel Temperature Anomaly (TSAH) in Aug–Oct partial correlations occurred early then peaked in mid-passage; for PSAH (Nov–March) correlations peaked at the end of passage. NAO and local temperatures (April–May) showed low correlations till late April, which then increased. For the Scandinavian Index (Jun–Jul) partial correlations peaked in mid-passage. Year was not selected in any of the best models, indicating that the climate variables alone accounted for Willow Warblers’ multiyear trend towards an earlier spring passage.

**Discussion:**

Climate indices for southern and eastern Africa dominated relationships in early spring, but western African indices dominated in mid- and late spring. We thus concluded that Willow Warblers wintering in southern and eastern Africa dominated early arrivals, but those from western Africa dominated later. We suggest that drivers of phenological shifts in avian migration are related to changes in climate at remote wintering grounds and at stopovers, operating with climate change in the north, especially for species with complex and long-distance migration patterns.

## Introduction

Many migrant birds have been arriving earlier for the European spring since about the 1980s; this has been attributed to climate change and gradually increasing springtime temperatures in Europe (*e.g.*, Sokolov et al., 1998; Hüppop & Hüppop, 2003; Vähätalo et al., 2004; Tøttrup, Thorup & Rahbek, 2006; Miles et al., 2017; Lehikoinen et al., 2019). But many long-distance Palaearctic migrants use wintering grounds that span Africa, for example Barn Swallow *Hirundo rustica*, European Reed Warbler *Acrocephalus scirpaceus*, Sedge Warbler *A. schoenobaenus*, Red-backed Shrike *Lanius collurio*, Spotted Flycatcher *Muscicapa striata*, Garden Warbler *Sylvia borin* and Willow Warbler *Phylloscopus trochilus* (Cepák et al., 2008; Fransson & Hall-Karlsson, 2008; Bairlein et al., 2014; Valkama et al., 2014). Migration is initiated thousands of kilometres from the breeding grounds and weeks before the birds arrive in Europe, so changing conditions in the wintering areas should also affect the timing of these long-distance migrants’ passage (Cotton, 2003; Gordo, 2007). The migrants’ responses to climate change at their northern European breeding grounds might result from a combination of different populations’ responses to conditions they had encountered at their respective winter quarters. Identifying the pattern of these relationships at multiple locations across widespread wintering areas is key to understanding a species’ response to climate change at its breeding grounds, because of carry-over effects of shifts in one life stage on phenology of the next stages in migrants (*e.g.*, Barshep et al., 2011; Tobolka et al., 2018; Tomotani et al., 2018).

Most studies have attributed the trends in the recent timing of the migrants’ arrival in Europe and North America to the increase in spring temperatures at their northern stopovers and breeding grounds (*e.g.*, Sokolov et al., 1998; Cotton, 2003; Hüppop & Hüppop, 2003; Miller-Rushing et al., 2008; Wood & Kellermann, 2015; Miles et al., 2017; Usui, Butchart & Phillimore, 2017; Zaifman et al., 2017; Lehikoinen et al., 2019; Redlisiak, Remisiewicz & Mazur, 2021). Conditions at the breeding grounds have been reported to drive phenological changes in the breeding of long-distance migrants more than conditions at the non-breeding grounds (*e.g.*, Ockendon, Leech & Pearce-Higgins, 2013). Yet conditions in the non-breeding season influence the migrants directly or indirectly, including carryover effects on spring migration by influencing overwinter survival and the: (1) timing of departure from the wintering grounds, (2) birds’ condition on departure, (3) duration and frequency of stopovers, (4) size of populations arriving from the wintering grounds (overviews in Gordo, 2007 and Remisiewicz & Underhill, 2020). A few studies have related migrants’ spring arrivals in Europe to conditions at non-breeding areas, including temperature and rainfall at migration stopovers (*e.g.*, Gordo, 2007; Saino et al., 2007; Saino & Ambrosini, 2008; Tøttrup et al., 2012; Halupka et al., 2017; Haest, Hüppop & Bairlein, 2020) and at the wintering grounds (*e.g.*, Katti & Price, 1999; Cotton, 2003; Salewski et al., 2004; González-Prieto & Hobson, 2013; Ouwehand & Both, 2017; Redlisiak, Remisiewicz & Nowakowski, 2018; Tobolka et al., 2018; Remisiewicz et al., 2019; Haest, Hüppop & Bairlein, 2020; Tomotani et al., 2021). Most studies on long-distance migrants, however, have focused on relationships between their spring arrival in Europe and conditions at their wintering grounds and stopovers in western Africa and southwestern Europe, reflected by the Sahel Precipitation Index (*e.g.*, Zwarts et al., 2009; Tobolka et al., 2018), precipitation and temperatures in the Sahel and in Europe (*e.g.*, Saino et al., 2007; Gordo & Sanz, 2008; Tøttrup et al., 2010; Haest, Hüppop & Bairlein, 2020), the Northern Atlantic Oscillation Index (NAO) in winter (*e.g.*, Forchhammer, Post & Stenseth, 2002; Cotton, 2003; Ahola et al., 2004; Gordo, Barriocanal & Robson, 2011; Haest, Hüppop & Bairlein, 2018), and the Normalized Difference Vegetation Index (NDVI) (*e.g.*, Balbontín et al., 2009; Tøttrup et al., 2010; Jørgensen et al., 2016; Thorup et al., 2017; Tomotani et al., 2021). Fewer studies have shown that the spring arrival of these migrants in Europe is also related to conditions in eastern and southern Africa, reflected by temperature (*e.g.*, Cotton, 2003); NDVI (*e.g.*, Tøttrup et al., 2012), the Southern Oscillation Index (SOI/ENSO) (*e.g.*, Stenseth et al., 2003; Cotton, 2003), and the Indian Ocean Dipole (IOD) (*e.g.*, Hušek et al., 2008; Tryjanowski, Stenseth & Matysioková, 2013; Tobolka et al., 2018). Most studies showing how climate at the non-breeding grounds influences the spring arrival of migrants have focused on locations in western Europe, where most migrants arrive from western Africa (Cepák et al., 2008; Fransson & Hall-Karlsson, 2008; Zwarts et al., 2009; Bairlein et al., 2014; Valkama et al., 2014). Few studies have shown the effects of climate change using indices of climatic or vegetation variation from both hemispheres (Cotton, 2003; Altwegg et al., 2012; Ockendon, Leech & Pearce-Higgins, 2013; Bussière, Underhill & Altwegg, 2015; Chambers et al., 2017; Tomotani et al., 2021). Even fewer authors have demonstrated the combined influences of some climate indices and temperatures in different parts of Africa on the arrival of species breeding in Europe (Cotton, 2003; Tobolka et al., 2018). In contrast to those studies, in an earlier study we showed that the timing of Willow Warblers’ arrival in northern Europe in spring is explained by a combination of at least seven climate indices that operate in different parts of Africa and in Europe (Remisiewicz & Underhill, 2020). We thus suggest that the spectrum of climate factors shaping spring arrivals in Europe for species with winter ranges that span most of Africa has been largely under-represented. In this study we therefore set out to examine in more detail the combined effects of a wide range of climate factors that affect Willow Warblers’ annual cycle.

The species winters throughout Africa south of the Sahara Desert and two subspecies migrate across the Polish coast of the Baltic Sea past the bird ringing station at Bukowo, Poland (54°20′–54°27′N, 16°14′–16°24′E), so we chose Willow Warblers as a representative long-distance migrant to determine how widespread patterns of climate might influence the timing of migration in complex ways. The two subspecies tend to occupy different winter quarters in Africa, but both breed in northern Europe (Bensch, Bengtsson & Åkesson, 2006; Bensch et al., 2009; Zhao et al., 2020); thus we expected that the pattern of their spring arrivals at stopovers on the Baltic Sea coast might be related to climate factors operating in different areas in Africa.

*Ph. t. trochilus* arrives at its breeding grounds in central Sweden from winter quarters in west Africa before *Ph. t. acredula* from eastern and southern Africa (Hedenström & Pettersson, 1984). These subspecies’ migration routes overlap at Bukowo (Remisiewicz & Underhill, 2020), which is ca 600 km south of their hybridisation zone in central Sweden (Bensch, Bengtsson & Åkesson, 2006; Bensch et al., 2009). We expected that the species’ spring passage through Bukowo would be a continually changing mixture of populations from different wintering quarters in Africa, possibly in a consistent pattern. Thus we investigated whether the timing of the early, middle and late cohorts of Willow Warblers at Bukowo during spring passage could be related to climate indices operating in different parts of their winter range. We also examined whether the timing of the spring passage at Bukowo was related to the numbers of juvenile Willow Warblers that had migrated through the site the previous autumn, using these numbers as a proxy for breeding success. Additionally, we aimed to test if a temporal decomposition of the Annual Anomaly (AA) of migration that we had developed (Remisiewicz & Underhill, 2020) helped to identify any temporal patterns in the relationships between consecutive periods of the AA and different climate indices.

## Materials and Methods

### Study species

**Figure 1:**
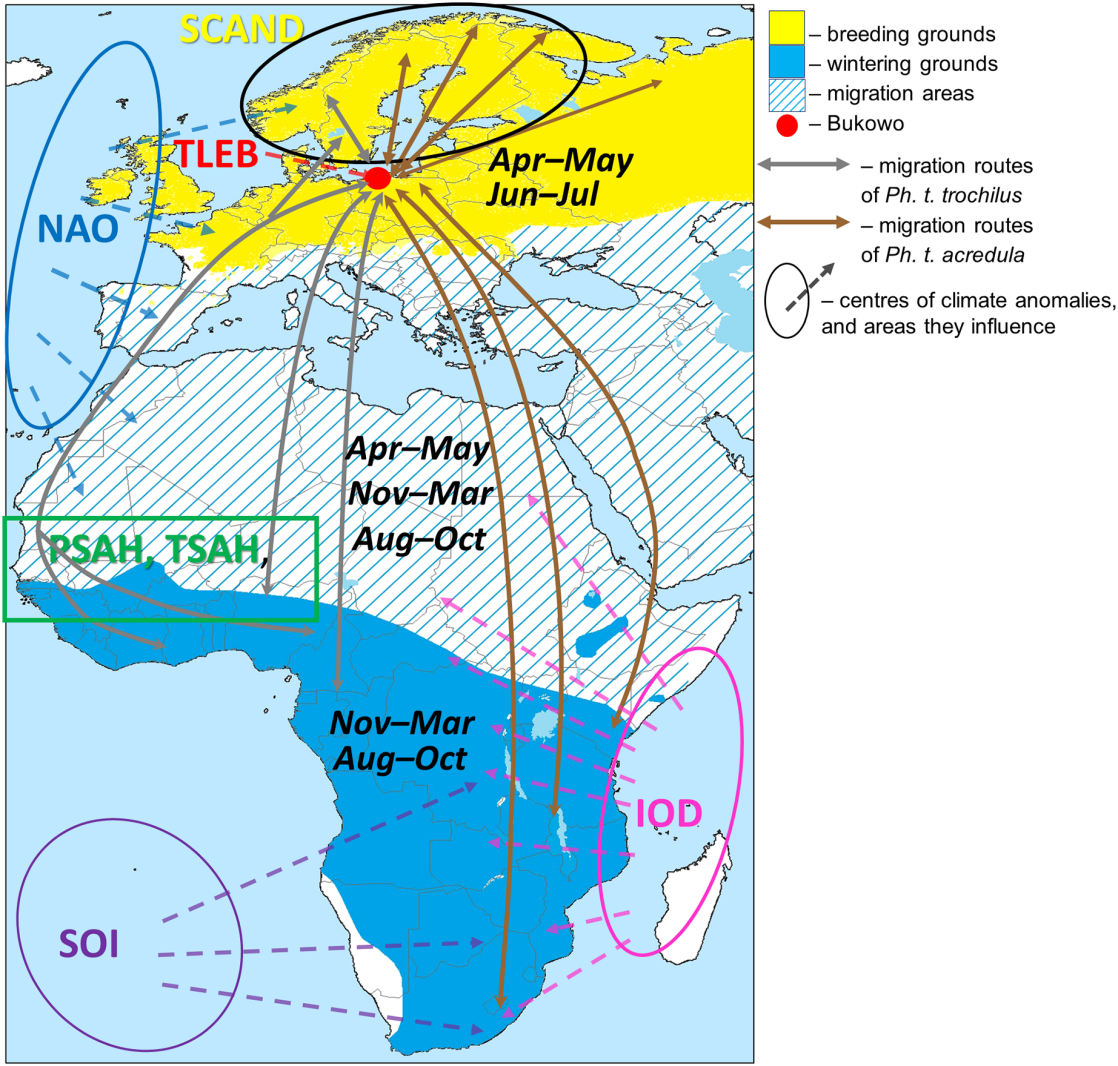
Approximate migration routes of Willow Warblers that pass through Bukowo and areas influenced by the large-scale climate variables. Symbols of the climate indices and the sources we used to visualise the regions they influence are presented in Table 1. The ranges of months are the main periods when Willow Warblers occur within their breeding, migration and wintering areas. The westernmost route in the Sahel region reflects geolocator tracks of birds breeding in Denmark (Lerche-Jørgensen et al., 2017), the eastern route considers genotyping (Zhao et al., 2020). This figure is based on Remisiewicz & Underhill (2020, modified), and is derived from “*Phylloscopus trochilus* Range Map.png” by Keith W. Larson, licensed under CC-BY-SA-3.0 by Magdalena Remisiewicz.

Two subspecies of Willow Warbler, *Ph. t. trochilus* and *Ph. t. acredula*, migrate through the region around the Baltic Sea to breeding grounds in northwestern Europe (Remisiewicz & Underhill, 2020), where they meet at a migratory divide (Fig. 1) (Tomiałojć & Stawarczyk, 2003; Bensch, Bengtsson & Åkesson, 2006; Bensch et al., 2009). In spring *Ph. t. trochilus* migrates ca 5,000 km from winter quarters mainly in west and central Africa south of the Sahara to breeding grounds in southern Scandinavia, Poland, and western and southern Europe; *Ph. t. acredula* flies 5,000–12,000 km from wintering grounds mainly in central, eastern and southern Africa to breeding grounds in northern Sweden, Finland, eastern Poland and far northeastern Europe (Fig. 1) (Hedenström & Pettersson, 1987; Cramp & Brooks, 1992; Bensch, Andersson & Åkesson, 1999; Tomiałojć & Stawarczyk, 2003; Bensch, Bengtsson & Åkesson, 2006; Fransson & Hall-Karlsson, 2008; Bensch et al., 2009; Zwarts et al., 2009; Valkama et al., 2014; Lerche-Jørgensen et al., 2017; Maciąg et al., 2017; Zhao et al., 2020). The proportion of Willow Warblers that winter in eastern and southern Africa increases along a west-east axis across the breeding grounds (Zwarts et al., 2009). *Ph. t. trochilus* arrives in the region of the migratory divide at 60°00′–63°00′N in central Sweden (Fig. 1) in April, about two weeks earlier than *Ph. t. acredula* (Hedenström & Pettersson, 1984). The sequence and proportions of each subspecies that migrate through Bukowo are difficult to estimate because the subspecies differ only subtly in colour and size (Bensch et al., 2009). These slight differences are further confounded by size differences between the sexes (Svensson, 1992).

**Table 1:** Explanatory variables used in modelling Willow Warbler spring migration (1 April–15 May) over 1982–2017 at Bukowo, Poland.

No	Symbols used in the text	Explanatory variable	Source, key references
1	**TLEB APR–MAY**	Apr–May mean of daily means of local temperatures in Łeba	http://www.ecad.eu
2	**NAO APR–MAY**	Apr–May mean of Northern Atlantic Oscillation Index	http://www.cpc.ncep.noaa.gov/ Hurrell (1995)
3	SCAND APR–MAY	Apr–May mean of Scandinavian Index	http://www.cpc.ncep.noaa.gov/ Bueh & Nakamura (2007)
4	**NAO NOV–MAR**	Nov–Mar mean of Northern Atlantic Oscillation Index	http://www.cpc.ncep.noaa.gov/ Hurrell (1995)
5	**PSAH NOV–MAR**	Nov–Mar mean of Sahel Precipitation Index within 10^o^–20^o^N, 20^o^W–10^o^E	http://research.jisao.washington.edu/data/sahel//sahelprecip19012017 Munemoto & Tachibana (2012)
6	TSAH NOV–MAR	Nov–Mar mean of Sahel temperature anomaly within 10^o^–20^o^N, 20^o^W–10^o^E	http://climexp.knmi.nl ERA5 dataset
7	IOD NOV–MAR	Nov–Mar mean of Indian Ocean Dipole	https://psl.noaa.gov/gcos_wgsp/Timeseries Marchant et al. (2006)
8	SOI NOV–MAR	Nov–Mar mean of Southern Oscillation Index	http://www.cpc.ncep.noaa.gov/ Richard et al. (2001)
9	NAO AUG–OCT_1y	Aug–Oct mean of previous year’s Northern Atlantic Oscillation Index	http://www.cpc.ncep.noaa.gov/ Hurrell (1995)
10	**PSAH AUG–OCT_1y**	Aug–Oct mean of previous year’s Sahel Precipitation Index	http://research.jisao.washington.edu/data/sahel//sahelprecip19012017
11	**TSAH AUG–OCT_1y**	Aug–Oct mean mean of previous year’s Sahel temperature anomaly within 10^o^–20^o^N, 20^o^W–10^o^E	http://climexp.knmi.nl ERA5 dataset
12	**IOD AUG–OCT_1y**	Aug–Oct mean of previous year’s Indian Ocean Dipole	https://psl.noaa.gov/gcos_wgsp/Timeseries; Marchant et al. (2006)
13	**SOI AUG–OCT_1y**	Aug–Oct mean of previous year’s Southern Oscillation Index	http://www.cpc.ncep.noaa.gov/ Richard et al. (2001)
14	NAO JUN–JUL_1y	Jun–Jul mean of previous year’s Northern Atlantic Oscillation Index	http://www.cpc.ncep.noaa.gov/ Hurrell (1995)
15	**SCAND JUN–JUL_1y**	Jun–Jul mean of previous year’s Scandinavian Index	http://www.cpc.ncep.noaa.gov/ Bueh & Nakamura (2007)
16	Year	Year as a number; 1982 = Year 1	Our database

**Note:**

The nine main climate indices used in the second step of analyses are marked in bold.

Willow Warblers arrive in northwestern Europe in April–May, breed in June–July, migrate south in August–October, then stay in Africa in November–March (Cramp & Brooks, 1992; Herremans, 1999; Tomiałojć & Stawarczyk, 2003; Dean, 2005; Zwarts et al., 2009; Kluen, Nousiainen & Lehikoinen, 2017). So we considered April–May to be “spring migration”, August–October to be “autumn migration” and “winter” to be November–March.

### Study site and sampling

We used daily numbers of Willow Warblers ringed during spring migration (1 April–15 May) in 1982–2017 using standardised protocols at the Bukowo ringing station on the Baltic Sea coast (54°20′13″–54°27′11″N, 16°14′36″–16°24′08″E) (Table S1, Supplemental Information). This was the same dataset (Supplement 2) as used in our previous paper (Remisiewicz & Underhill, 2020), but here we analysed the data in greater detail and processed it differently to determine any changes in the influence of each climate index over spring passage. We also used the total numbers of juvenile Willow Warblers caught at Bukowo each autumn migration (14 August–29 October) in 1981–2016. Migrating passerines were caught in mist nets placed in mixed coastal and riparian forests and bushes on spits between the Baltic Sea and the neighbouring Bukowo and Kopań coastal lakes (Busse & Meissner, 2015; Nowakowski et al., 2021). In 1982–2010 ringing was conducted on the spit of Lake Kopań (54°27′11″N, 16°24′08″E), but then holiday housing was developed nearby and human disturbance increased. So in 2012–2017 ringing operations were moved 16 km east to the quiet spit of Lake Bukowo (54°20′13″N, 16°14′36″E). Both locations occupy similar coastal habitats on narrow spits of coastal lakes that channel migrating birds’ passage, so we combined the results from both sites into one dataset (“Bukowo”). The number of 8 m-long nets was stable within each season, but ranged from 35 to 57 in spring, and from 38 to 76 in autumn in different years (Table S1). Ringing was conducted each year from 23 March–15 May and from 12 August–1 November. This extended beyond the Willow Warblers’ passage in spring (1 April–15 May) and in autumn (14 August–29 October). In several springs ringing was prolonged beyond the standard period and mostly local Willow Warblers were caught after 15 May, as indicated by local recaptures (Supplement 2). Birds caught in some autumns before 14 August were also mostly local breeders (Supplement 2). Birds were caught between dawn and dusk, ringed, measured, aged, and then released (Busse & Meissner, 2015). In spring all Willow Warblers were in the same plumage and were aged as “full grown”; in autumn they were aged as juveniles or adults (Svensson, 1992; Demongin, 2016). We counted an individual only at the first capture in each season. The fieldwork was conducted with the annual approval of the General Directorate for Environmental Protection, Poland, supported by the Polish Academy of Sciences (last decision: DZP-WG.6401.03.97.2017.jro), and the Marine Office, Słupsk (last decision: OW-A-510/87/17/ds).

### Climate indices

We used 15 large-scale and local climate indices for the Willow Warbler wintering grounds in western, eastern and southern Africa and for their migration routes through Europe (Fig. 1, Table 1). The large-scale climate indices reflect rainfall and temperatures over wide areas (Wang & Schimel, 2003; Fig. 1). For the Sahel region, where no large-scale climate index was available, we used the Sahel Precipitation Index (PSAH) and the Sahel temperature anomaly (TSAH) within 10–20°N and 20°W–10°E (Fig. 1). We also used local temperatures in Łeba (TLEB), the closest coastal weather station to Bukowo. We downloaded monthly values for these climate indices from the websites of weather services and visualised their influence based on these sources and the literature (Table 1). Negative summer values of the Scandinavian index (SCAND) are related to high rainfall and low temperatures over Scandinavia (Bueh & Nakamura, 2007). The positive phase of the North Atlantic Oscillation Index (NAO) in winter is related to warm and wet winters in western Africa and early springs in western Europe (Hurrell, 1995). The positive winter Indian Ocean Dipole (IOD) is related to high rainfall in eastern and southeastern Africa (Marchant et al., 2006), and usually varies independently of the Southern Oscillation Index (SOI) (Ashok, Guan & Yamagata, 2003). A negative winter SOI indicates El Niño conditions, related to dry and hot weather across southern Africa but warm and wet weather in eastern Africa; a positive winter SOI indicates La Niña conditions, which has the opposite effect of an El Niño on climate in southern and eastern Africa, with a weak relation to cool conditions in western Africa south of the Sahel (Richard et al., 2001; McPhaden, Santoso & Cai, 2020). The monthly Sahel Precipitation Index (PSAH) and the Sahel Temperature Anomaly (TSAH) reflect the anomalies of these climate features within the western Sahel where Willow Warblers overwinter (Fig. 1, Table 1). Negative monthly values of PSAH indicate low precipitation in relation to the 1980–2009 baseline. Negative monthly values of TSAH indicate high temperatures in relation to the 1982–2017 baseline. These indices were used as proxies for the conditions that the Willow Warblers experience during consecutive periods of their life (Fig. 1). Thus we averaged each climate index, including PSAH, TSAH and TLEB, for April–May (spring migration), November–March (wintering), August–October of the previous year (autumn migration), and June–July the previous year (breeding). For further analyses we selected the climate indices in the regions where Willow Warblers occur, in the months we examined, as in our previous study (Remisiewicz & Underhill, 2020). For each climate index we used only the periods when Willow Warblers occur within the area influenced by that index in the year before their spring migration (Fig. 1). For example, we used the indices of climate such as PSAH, TSAH, IOD and SOI that cover Willow Warblers’ non-breeding grounds for August–October and November–March, the periods when we expected the birds to occur in Africa where these indices operate, but not for April–May and June–July, when the birds should be on spring passage or at the breeding grounds in Europe. By these criteria we selected 15 climate variables for further analysis (Table 1). We used these 15 indices and the year (Table 1) as explanatory variables in multiple regression models to identify their combined effects on the yearly timing of Willow Warbler spring migration across the southern Baltic Sea coast. We avoided using correlated climate variables and we monitored Variance Inflation Factors (VIF) in our multiple regression models (Dormann et al., 2013) to mitigate any influence of multicollinearity on our results, as in Remisiewicz & Underhill (2020).

## Statistical analyses

### Selection of data on spring and autumn migration at Bukowo

We analysed the numbers of Willow Warblers caught each day between 1 April and 15 May at Bukowo in 1982–2017, but excluded 1993 and 2011, years when fewer than 30 Willow Warbler were caught. Our dataset thus included 34 spring seasons with more than 30 individuals captured (Table S1). These daily totals were transformed to daily proportions (percentages) of the total number of this species caught that spring. The percentages were summed to produce a cumulative arrival curve for the season so that each year had an equal weight in generating the multiyear arrival curve. We also used the total numbers of juvenile Willow Warblers caught at Bukowo in the previous autumn migration (14 August–29 October) in 1981–2016 as a measure of the species’ breeding success north and northeast of Bukowo. To account for any differences in catching effort between autumns, we calculated the number of juveniles caught each autumn per 50 mist nests, by dividing the total of juveniles caught each autumn by the number of mist nets used that season, and multiplying this index by 50 (Table S1). The numbers of Willow Warblers did not differ between the Kopań and Bukowo ringing sites, either in each season’s totals or in the numbers of birds caught per mistnet, in spring and in autumn (Table S3); thus we combined the data from these locations in one multiyear dataset.

### Methods of calculating the Annual Anomaly and its temporal decomposition

**Figure 2:**
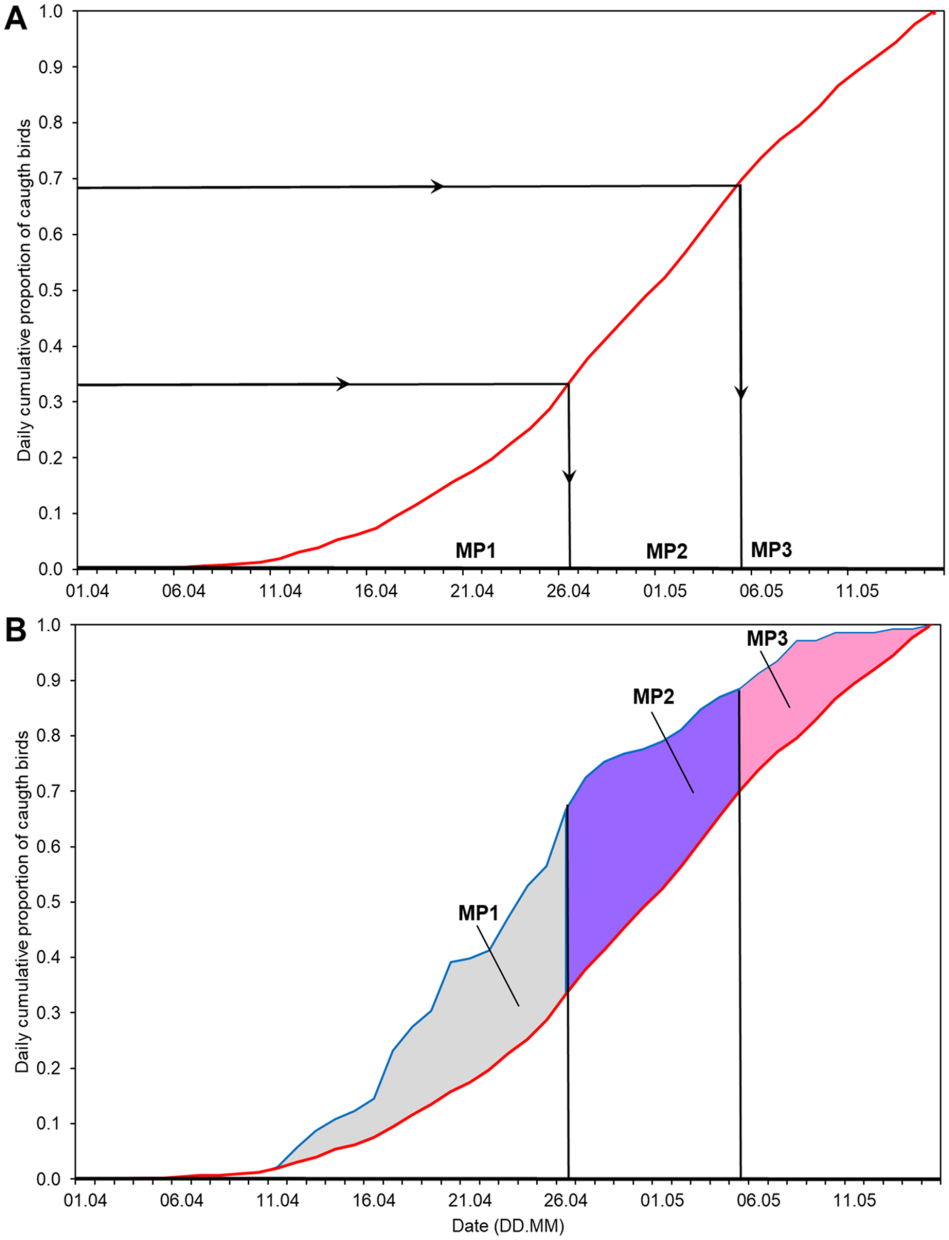
Division of the overall spring migration curve into thirds of average passage 1982–2017 and the division of Annual Anomaly (AA) for 2012 into three main periods using the derived ranges of dates. (A) Division of the multiyear average migration curve into three non-overlapping main periods of spring (MP1, MP2, MP3). Red line = 1982–2017 average migration curve, black lines = division into thirds of average passage. (B) Ranges of dates from Fig. 2A to decompose AA for 2012 into three periods. Blue line = migration curve for 2012. The areas in the three main periods (blue and white patterns), total to overall AA for 2012. MP1, main period 1; MP2, main period 2; MP3, main period 3; ranges of percentiles and symbols of periods as in Table 2.

We calculated the time series of the annual anomaly (AA) for the whole spring in relation to the average cumulative curve of spring passage in 1982–2017 (Fig. 2), as in Remisiewicz & Underhill (2020). Then we selected the dates of the thirds of passage (0–33%, 34–66% and 67–100% of Willow Warblers caught; Table 2) of the average spring migration derived from the overall multiyear cumulative curve (Fig. 2). We used these dates to decompose the annual anomaly each spring into three non-overlapping main periods (Fig. 2, Table 2). The three time series formed in this way reflect the year-to-year changes in passage within each of the three main periods of the spring seasons over the years we studied. First, we used these three time series as explanatory variables, along with the year and the number of birds caught each spring, in multiple regression to test for any potential effect of bird numbers on the arrival dates (Tryjanowski & Sparks, 2001). Then we checked for any trends over 1982–2017 in these three time series using linear regression, applying the Benjamini-Hochberg correction for multiple comparisons (Benjamini & Hochberg, 1995).

**Table 2:** Sections of the Annual Anomaly (AA) for main periods and sub-periods of spring, and for the whole spring, used as response variables in modelling Willow Warbler spring migration (1 April–15 May) in 1982–2017 at Bukowo, Poland.

No	Symbol used in the text	Percentiles	Response variable
0	AA	0–100%	Annual Anomaly for 1 April–15 May
**1**	**MP1**	**0–33%**	**Annual Anomaly for 1–26 April**
**2**	**MP2**	**34–66%**	**Annual Anomaly for 27April–5 May**
**3**	**MP3**	**67–100%**	**Annual Anomaly for 6–15 May**
1	SP1	0–20%	Annual Anomaly for 1–22 April
2	SP2	11–30%	Annual Anomaly for 18–25 April
3	SP3	21–40%	Annual Anomaly for 24–28 April
4	SP4	31–50%	Annual Anomaly for 26 April–1 May
5	SP5	41–60%	Annual Anomaly for 29 April –3 May
6	SP6	51–70%	Annual Anomaly for 2–5 May
7	SP7	61–80%	Annual Anomaly for 4–7 May
8	SP8	71–90%	Annual Anomaly for 6–11 May
9	SP9	81–100%	Annual Anomaly for 9–15 May

**Note:**

Percentiles = the ranges of percentiles at multiyear curve used to derive the ranges of dates used to distinguish main periods (MP) and sub-periods (SP) of AA (Fig. 2). Three main non-overlapping periods of spring are marked in bold.

### Multiple regression models for periods of AA

As in our previous study (Remisiewicz & Underhill, 2020), we standardized response and explanatory variables so that they had a mean of 0 and a standard deviation of 1. This scaling does not influence the statistical significance of the results but does aid interpretation. We modelled each of the three main periods as a response variable and used the 15 large-scale climate indices and the year as 16 explanatory variables (Table 1) in multiple regression models using the R package “MuMIn 1.43.6” (Bartoń, 2019), as we did with overall AA in the previous study (Remisiewicz & Underhill, 2020). We retained year as an explanatory variable to control for its effect while modelling the effects of the climate variables (Frost, 2019); if it was not significant in the models that were finally selected, we could infer that the long-term trend had been subsumed into the climate variables. We used the explanatory variables as linear functions of AA because they showed stronger linear relationships with the overall AA than when used as polynomial terms (Remisiewicz & Underhill, 2020). We used “all subsets regression” and calculated the Akaike Information Criteria corrected for small sample size (AICc) for each subset of the explanatory variables, then we selected the best model using model ranking by AICc with the package “MuMIn 1.43.6” (Bartoń, 2019). The adjusted coefficient of determination (AdjR^2^) measured the proportion of variation explained by the best models for each of the spring periods. We calculated partial correlation coefficients (*pR*) for each best model using the package “ppcor 1.1” (Kim, 2015). The *pR* reflects the direction and the strength of the correlation between the response variable and each explanatory variable while removing the effects of the remaining variables. The statistical analyses were conducted in R 4.0.3 (R Core Team, 2020).

Motivated by our results, we applied the same approach to a series of nine overlapping sub-periods of the AA (Table 2). Each sub-period spanned 20% of the the multiyear curve (0–20%, 10–30%, …, 80–100%; Table 2), and we used the dates for these ranges from the multiyear cumulative curve to divide the AA for each spring into the nine sub-periods, in the same way we initially split the passage period in thirds (Fig. 2). In the Results, we show that nine of the 15 candidate climate indices were selected in one or more of the three best-fitting models for the three main periods of the AA and contributed most in the top models (ΔAIC_c_ < 2). We investigated the influence of these nine explanatory variables throughout the spring migration over the nine sub-periods. We then produced a graphical display that indicated the importance of each explanatory variable as it waxed or waned through spring passage by plotting the sequential partial correlation coefficients *pR* for the nine explanatory variables. The nine sub-periods overlapped, so these results cannot be interpreted as a series of formal statistical models. The plots are intended to be interpreted as visual and diagnostic displays, in the spirit of the exploratory approach to data analysis pioneered by Tukey (1980). It is helpful to think of this approach as an extension of the concept of the moving average, a similar “windowing” approach used in descriptive time series analysis to visualize patterns through time.

### Analysing relationships between spring migration and the number of juveniles caught the previous autumn at Bukowo

To explore any potential effect of seasonal breeding success on the Willow Warblers’ migration timing the following spring, we used linear regression to model the relationship between the overall Annual Anomaly and its three main periods at Bukowo each year in 1982–2017 and the count of juveniles captured at the site in the previous autumn over 1981–2016 (Njuv_1y). The number of juveniles caught in autumn 1982 was nearly five times larger than the mean numbers caught in other autumns though the numbers and locations of mist nets were similar each year (Table S1). This exceptional number possibly arose from an unusual weather pattern that season, which might have changed the species’ migration corridor across the Baltic, so we excluded this season from our analysis. Thus we used 33 years of data for this analysis, a different dataset from that used for spring. In the preliminary multiple regressions that included the count of juveniles this explanatory variable was never selected in any of the top models (ΔAIC_c_ < 2), likely because its effect was weaker than the effects of the climate variables. We therefore did not include the count of juveniles in our final multiple regression models but analysed this relationship separately. The distribution of the count of juveniles departed from normal, so we applied a square root transformation to standardise this data and used the output as the explanatory variable in regression analyses.

## Results

### Differences in the long-term trends of Willow Warbler migration timing in three main non-overlapping periods of spring

**Figure 3:**
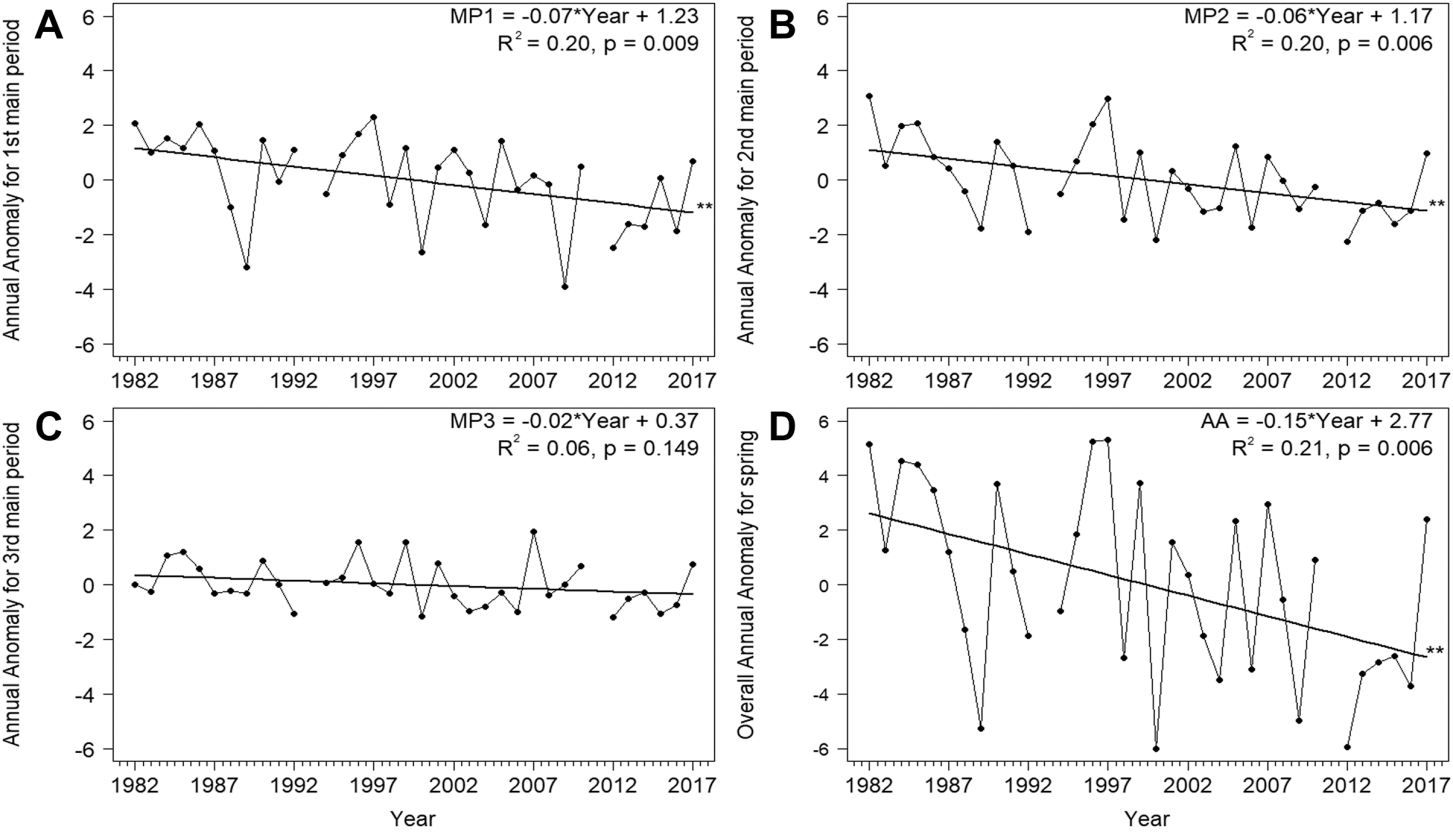
Trends for the Annual Anomaly in three main periods (A)–(C) and the whole season (D) for Willow Warbler spring migration at Bukowo, Poland, 1982–2017. Symbols of spring periods as in Table 2 and Fig. 2; **–*p* significant after Benjamini-Hochberg correction for multiple comparisons. More statistics for regression equations in Table S7.

Based on the mean overall cumulative curve of the numbers of Willow Warblers caught in spring over 1982–2017, the 0–33% range of passage spanned 1–26 April, 34–66% spanned 27 April–5 May, and 67–100% spanned 6–15 May (Fig. 2A). So we divided AA into three main periods in each year using these dates (Fig. 2B, Table 2). The numbers of birds caught each spring had no significant influence on anomalies in these three periods and on the overall AA (Table S3). Next we analysed the trends of these time series against the year (Fig. 3). The passage of Willow Warblers within the first main period shifted earlier by 2.4 days on average over 1982–2017 (Fig. 3A). The passage in the second period shifted earlier by 2.3 days on average over those years (Fig. 3B). These shifts jointly contributed to 87% of the shift in the overall anomaly of spring passage over the 36 years of our study (Fig. 3, Table S7). The shift of 0.7 days earlier on average for the last period of spring was not significant (Fig. 3C). The overall timing of Willow Warbler spring migration at Bukowo over 1982–2017, reflected by the overall Annual Anomaly, advanced on average by 5.4 days, a sum of the shifts in all three main periods (Fig. 3D; Remisiewicz & Underhill, 2020). The linear regression, although significant, explained only 20% and 21% of the variation in the first two main periods of spring (Figs. 3A, 3B, Table S7), so we then examined other sources of year-to-year variation besides these multiyear trends.

### The relationships between large-scale climate indices and Willow Warbler migration in three main periods of spring

In our earlier study (Remisiewicz & Underhill, 2020) we identified a set of climate variables that explained year-to-year variation in the overall Annual Anomaly (AA) of Willow Warbler spring migration at Bukowo in 1982–2017; here we explore the influences of these climate variables on the timing of the species’ spring migration at a finer resolution by examining the three main periods of spring. The anomaly of passage in the first period (MP1) was significantly negatively related to six climate variables selected by the best model, which explained AdjR^2^ = 56.6% of its variation (Table 3; full model in Table S8, model selection in Table S9, model diagnostics in Fig. S2). The best model for the middle period (MP2) explained AdjR^2^ = 66.4% of its variation by a negative relationship to each of five climate variables (Table 3, Table S10, Fig. S3). The anomaly of the last period (MP3) was negatively related to each of five climate indices, as indicated by the best model, which explained 30.7% of its variation (Table 3, Table S11, Fig. S4). All these relationships were negative (Table 3), meaning an earlier passage with a higher climate index and *vice versa*. Nine climate variables, which were selected in the best models for at least one of the three main periods (Table S5) and contributed at least 60% in any of the three sets of top models with ΔAICc < 2 (Table S13), were used for the multiple regressions of the nine sub-periods of spring. For pairs of highly correlated climate variables, for example SOI (Aug–Oct_1y) and (Nov–Mar) (Table S5), we chose the variable that contributed most to the top models (Table S13), to avoid multicollinearity.

**Table 3:** Relationship between climate variables and Annual Anomalies for the three main periods of spring (MP1–MP3), and for whole season (AA) of Willow Warbler spring migration at Bukowo, Poland, in 1982–2017.

Explanatory variable	Estimate	SE	*t*	*p*	VIF	*R* ^*2*^	*pR*
**MP1**	Best model statistics: F_6,28_ = 8.23, AdjR^2^ = 56.6%						
NAO APR–MAY	–0.39	0.12	–3.20	**0.003**	1.12	0.27	–0.52
SOI NOV–MAR	–0.46	0.13	–3.48	**0.002**	1.37	0.30	–0.55
IOD AUG–OCT_1y	–0.44	0.14	–3.04	**0.005**	1.63	0.25	–0.50
PSAH AUG–OCT_1y	–0.39	0.13	–2.94	**0.007**	1.38	0.24	–0.49
TSAH AUG–OCT_1y	–0.31	0.12	–2.46	**0.020**	1.22	0.18	–0.42
SCAND JUN–JUL_1y	–0.27	0.13	–2.13	**0.042**	1.22	0.14	–0.37
**MP2**	Best model statistics: F_5,29_ = 14.42, AdjR^2^ = 66.4%						
TLEB APR–MAY	–0.48	0.11	–4.22	**0.000**	1.28	0.38	–0.62
NAO APR–MAY	–0.48	0.10	–4.70	**0.000**	1.06	0.43	–0.66
IOD AUG–OCT_1y	–0.32	0.11	–2.91	**0.007**	1.20	0.23	–0.48
PSAH AUG–OCT_1y	–0.24	0.11	–2.26	**0.032**	1.18	0.15	–0.39
SCAND JUN–JUL_1y	–0.51	0.11	–4.52	**0.000**	1.31	0.41	–0.64
**MP3**	Best model statistics: F_5,29_ = 4.01, AdjR^2^ = 30.7%						
TLEB APR–MAY	–0.33	0.16	–2.14	**0.041**	1.20	0.14	–0.37
NAO APR–MAY	–0.25	0.15	–1.70	0.100	1.06	0.09	–0.30
NAO NOV–MAR	–0.30	0.15	–2.05	**0.050**	1.04	0.13	–0.36
PSAH NOV–MAR	–0.35	0.15	–2.41	**0.022**	1.06	0.17	–0.41
SCAND JUN–JUL_1y	–0.41	0.16	–2.54	**0.017**	1.26	0.18	–0.43
**AA**	Best model statistics: F_7,27_ = 8.09, AdjR^2^ = 59.4%						
TLEB APR–MAY	–0.31	0.13	–2.39	**0.024**	1.38	0.17	–0.42
NAO APR–MAY	–0.38	0.12	–3.31	**0.003**	1.12	0.29	–0.54
NAO NOV–MAR	–0.45	0.14	–3.22	**0.003**	1.65	0.28	–0.53
TSAH NOV–MAR	–0.35	0.15	–2.40	**0.023**	1.70	0.18	–0.42
SOI AUG–OCT_1y	–0.37	0.14	–2.54	**0.017**	1.75	0.19	–0.44
IOD AUG–OCT_1y	–0.49	0.15	–3.27	**0.003**	1.86	0.28	–0.53
SCAND JUN–JUL_1y	–0.33	0.13	–2.58	**0.016**	1.38	0.20	–0.44

**Note:**

Estimate–coefficients from multiple regression, SE–standard error, *t, p*–*t*-test and significance of each estimate, *p* < 0.05 in **bold** face. VIF–variance inflation factor, *R^2^*–partial determination coefficient for each factor, * pR*–partial correlation coefficient. Abbreviations of climate variables as in Table 1, symbols of explanatory variables as in Table 2. Full models presented in Table S8, model selection presented in Tables S9–S12.

SOI and IOD significantly influenced the timing of migration in the first period of spring, but had no influence on passage in the last period (Table 3). PSAH (Aug–Oct_1y) influenced migration in the first two periods; TSAH affected the first period, but had no influence on the last period; PSAH (Nov–Mar) influenced the passage in the last period. SCAND (Jun–Jul_1y) influenced migration in all three main periods of spring.

For the overall AA, the best model explained 59.4% of the variation and selected seven climate variables, six of which were among the indices selected in the best models for the three periods of AA (Tables S5, S12, S13, Fig. S5). The effect of TSAH (Nov–Mar) on AA corresponded with the effect of TSAH (Aug–Oct_1y) on MP1 (Table 3). For all four best models, an inspection of the residuals (Figs. S2–S5) showed they met the assumptions of multiple linear regression (Crawley, 2013). All values of VIF < 10 (Table 3) indicated no harmful collinearity in any of these models (Dormann et al., 2013).

### Sequence of relationships between large-scale climate indices and the timing of migration in nine overlapping sub-periods of spring

**Figure 4:**
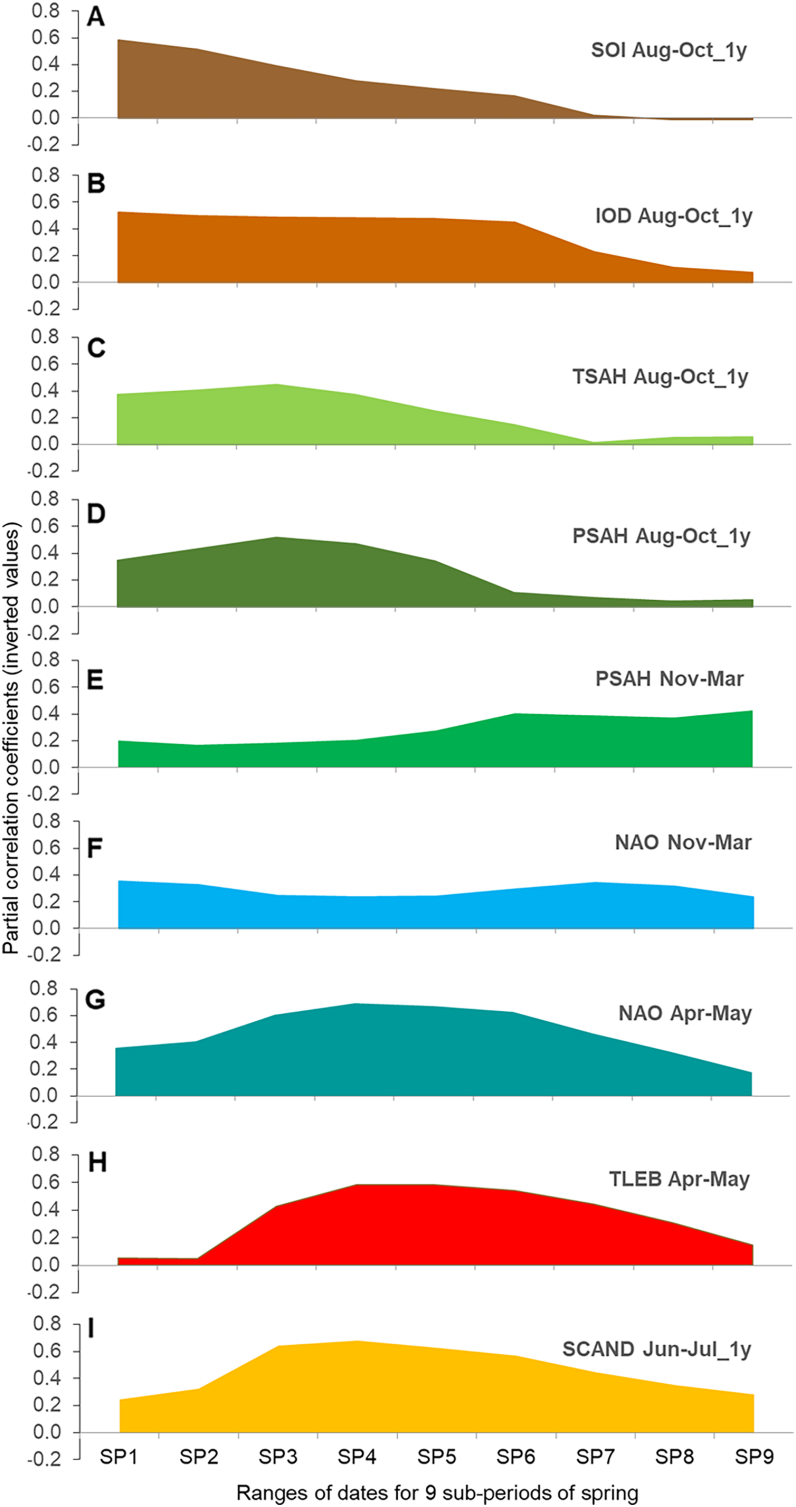
(A–I) Partial correlation coefficients for AA in nine overlapping sub-periods of spring against the nine main climate indices for Willow Warbler spring migration at Bukowo, Poland, 1982–2017. The signs of partial correlation coefficients (Table S14) were inverted, so the positive values at Y-axis indicate negative correlation to better visualise their changes with the progress of migration; source data in Table S15. Symbols of climate indices in Table 1, symbols of spring sub-periods in Table 2.

The regression models for each of the nine overlapping AA sub-periods against the nine selected explanatory variables suggested a gradual change in the influence of each climate index on the timing of Willow Warbler passage in subsequent periods of spring (Figs. 4A–4I, Tables S14, S15). The graphical display (Figs. 4A–4I) should be interpreted in conjunction with the formal models (Table 3, Table S14). Most of these relationships were negative (Table S14), indicating an earlier passage with higher values of the climate index, as for the three main periods (Table 3).

For the Southern Oscillation Index (SOI) and the Indian Ocean Dipole (IOD) in Aug–Oct of the previous year we found large correlations with the early and middle part of spring passage, which decreased in importance after mid-passage (Figs. 4A and 4B). The Sahel Temperature Anomaly (TSAH) in these months showed smaller partial correlations with the passage in the first half of spring, peaked in about mid-passage, before fading in importance (Fig. 4C). Similarly, the correlations for the Sahel Precipitation Index (PSAH) in Aug–Oct were moderate at the beginning of spring, peaked in mid-passage and then faded (Fig. 4D). PSAH in Nov–March showed low partial correlations in the first and the second periods of spring, but increased in the last part of spring migration (Fig. 4E). NAO in Nov–March showed low but consistent correlations over the whole passage (Fig. 4F). Partial correlations for NAO in Apr–May were moderate at the beginning of spring passage, peaked in the middle, then decreased at the end (Fig. 4G). The correlations for the local April–May mean temperatures in Łeba (TLEB Apr–May) were small at the beginning of spring, increased towards mid-passage, then decreased towards the end of migration (Fig. 4H). SCAND in Jun–Jul of the previous year showed moderate partial correlations for the first third of spring, peaked in mid-passage and remained important until the seasons’ end (Fig. 4I).

### Relationship between number of juveniles caught the previous autumn and the timing of spring migration

The SCAND index in June–July of the previous year, which reflects conditions in the previous breeding season in Scandinavia (Fig. 1), was related to the timing of Willow Warbler migration the following spring (Table 3, Fig. 4I, Table S14). We expected that a warm summer in Scandinavia might enhance breeding success, which should produce more juveniles migrating south in autumn, which in turn would likely influence the passage north the following spring. Thus we evaluated if the numbers of juvenile Willow Warblers migrating through Bukowo in autumn were related to the species’ migration timing in the following spring. So we checked if AA in each of the three main periods of spring was related to the number of juveniles caught the previous autumn. The numbers of juveniles caught in autumn thus served as a proxy for the Willow Warblers’ breeding success north and northeast of Bukowo (Fig. 1).

**Figure 5:**
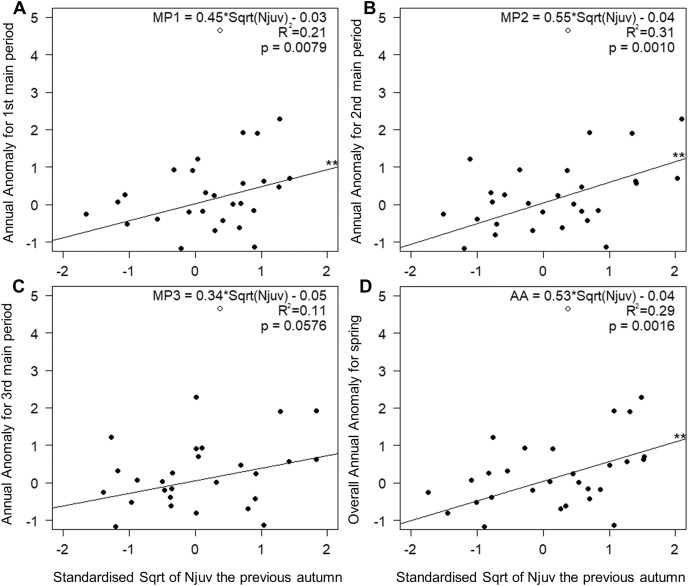
Relationships between AA in three main periods (A–C) and the whole season (D) of Willow Warbler spring migration in 1982–2017 and the count of juveniles caught the previous autumn at Bukowo, Poland. White circle = the outlier value in autumn 1982 excluded from the regression. **–*p* significant after Benjamini-Hochberg correction for multiple comparisons. Details of regressions in Table S16.

The number of juveniles in autumn, as the sole explanatory variable, accounted for 21% and 31% of the variation in the timing of the first and the middle main periods of spring passage (Figs. 5A and 5B). This relationship was not significant in the last third of spring (Fig. 5C), but did explain 29% of the variation in the overall passage (Fig. 5D). Migration through Bukowo, especially in the first and the middle periods of spring, occurred later the larger the numbers of juveniles caught in the previous autumn migration and *vice versa* (Fig. 5, Table S16). The number of juveniles in autumn at Bukowo was not correlated with the SCAND index in Jun–July of the same year (Pearson’s correlation: *r* = −0.08, *p* = 0.65).

## Discussion

Indices of climate in southern and eastern Africa (IOD and SOI) were related to the timing of Willow Warbler’s arrival at Bukowo in the early and middle of the three main periods of spring passage. The indices of climate in western Africa (TSAH, PSAH and NAO), showed weaker relationships to the first period, which increased in the middle and late periods. This pattern leads us to suggest that Willow Warblers from eastern and southern Africa arrive early at the southern coast of the Baltic Sea, where they mix with populations from western Africa, which mostly arrive later in the season. This sequence is the inverse of the arrival timing of the two subspecies of Willow Warblers at their breeding grounds in Sweden (Hedenström & Pettersson, 1984; Bensch, Bengtsson & Åkesson, 2006; Bensch et al., 2009), so we present possible explanations for this apparent discrepancy. We found that climate indices reflecting conditions at the wintering grounds and at stopover sites in Africa influenced migration timing at Bukowo more than local spring temperatures, which contradicted the findings of other studies (*e.g.*, Usui, Butchart & Phillimore, 2017; Lehikoinen et al., 2019). We explain how a combination of remote and local climate variables has enabled the pattern of earlier arrivals in spring and the earlier breeding in Europe that has been observed for many migrant birds since the 1980s. We found the long-term trend for Willow Warblers to arrive earlier in spring at Bukowo, as at many other locations in Europe (Remisiewicz & Underhill, 2020), was caused by an earlier passage in the first two main periods of spring, but not in the last period; we will discuss this novel insight.

### Long-term trends in the timing of Willow Warbler passage in three main sub-periods of spring

The spring migration of Willow Warblers at Bukowo over the 36 years of our study shifted earlier in the first two main periods of the season (1 April–5 May), but not in the third period (6–15 May). These combined changes led to a shift in the Annual Anomaly for the whole spring (Fig. 3). Willow Warblers migrate through Bukowo to breeding grounds in Sweden, where the population size decreased over 1975–2016 (Green, Haas & Lindström, 2017), to Finland where the population increased in this period (Finnish Museum of Natural History, 2017), and to other breeding grounds (Fig. 1). Birds from these breeding populations mix at Bukowo in unknown proportions, thus it was difficult to relate the numbers of birds caught at this station to population trends. The earlier arrival of Willow Warblers at Bukowo over the years we examined (Fig. 3) should be considered an indication of the extent of this phenomenon, rather than an absolute measure of the shift to an earlier spring passage. These shifts correspond with the advance in Willow Warbler arrivals observed over other years at other locations in Europe (*e.g.*, Sokolov et al., 1998; Cotton, 2003; Hüppop & Hüppop, 2003; Tøttrup, Thorup & Rahbek, 2006; Hedlund et al., 2015; Miles et al., 2017; Remisiewicz & Underhill, 2020). Other studies of long-distance migrants have shown larger shifts in the timing of early percentiles of the spring passage (5%, 10%) than for late percentiles (90%, 95%), as in our results. The earlier studies explained the pattern by showing a stronger effect on the beginning of migration than on its end of increased temperatures or other climate variables along migration routes and locally at bird observatories (Ahola et al., 2004; Jonzén et al., 2007; Miles et al., 2017; Lehikoinen et al., 2019). We suggest a more complex explanation for these temporal shifts of spring migration timing as a combined effect of two phenomena: (1) changes in climate factors, some with multiyear trends and others without, which have different effects on different cohorts of migrating Willow Warblers; (2) different selection pressures on early- and late-arriving male and female Willow Warblers.

Firstly, we suggest that the varied advance of the Willow Warbler passage at Bukowo in three main periods of spring was caused by the sequential passage of different migratory populations, which arrive from different parts of the wintering range where they had been affected by different climatic conditions. These populations then mix at the site in proportions that vary over the season. Of the nine climate variables that were related to the timing of the Willow Warblers’ passage through Bukowo (Table 3), the values of PSAH and TSAH (Aug–Oct_1y) and temperatures in Łeba (Apr–May) increased significantly over the 36 years of study (Table S6). PSAH and TSAH (Aug–Oct_1y) were related with the timing of migration in the first two main periods of spring (Table 3); these climate factors might therefore help explain the long-term trend towards an earlier passage in spring (Figs. 3A and 3B). Analogously, long-term increases in TLEB (Apr–May) likely contributed to the advance in the middle period of spring, along with PSAH (Fig. 3B). TLEB (Apr–May) was the only climate variable with a long-term increase that influenced the timing of the species’ passage in the last period of spring (Table 3), which by acting alone explained the smaller advance than in the other periods (Fig. 3C). Year as the only explanatory variable was significantly related to anomalies in the first and second main periods of spring (Fig. 3, Table S7). However when used in conjunction with the other climate variables, year was not selected in any of the best models (Table 3). This indicated that the effect of the year was absorbed by the climate variables that accounted for the long-term trends in the timing of migration. Other climate indices showed no clear trend, but their year-to-year changes contributed to the interannual variability in Willow Warblers’ spring migration timing, as in other migrants (*e.g.*, Tomotani et al., 2021).

Secondly, selection pressures might act differently on the sexes, especially in protandrous species in which males arrive at the breeding grounds ahead of the females. The selection pressures for early arrival vary between males and females (Kokko, 1999; Morbey & Yndenberg, 2001; Kokko et al., 2006; Spottiswoode, Tøttrup & Coppack, 2006; Lerche-Jørgensen et al., 2018). Protandry in Willow Warblers has increased in the Baltic region over 1990–2012 (Hedlund et al., 2015). The prevalence of males, pressed for early arrival, among the first spring migrants at Bukowo (Šoštarić, 2016) likely contributed to the shift towards an earlier passage in the first and the middle of the three main periods of spring that we found over 1982–2017.

The explanation for larger long-term shifts to an earlier passage in the first two periods of spring rather than in the third period likely lies within the combined influences of long-term increases in climate indices that reflect wetter or warmer winters and springs, which favour migrants’ survival and the selection pressure for early arrival, the evolutionary mechanism that drives the migrants’ adjustment to these changes (Kokko, 1999; Kokko et al., 2006).

### Temporal variation in the effects of climate indices on the timing of spring passage at Bukowo

*Ph. t. acredula* winters mainly in eastern Africa (Zhao et al., 2020), where IOD reflects rainfall and temperatures (Marchant et al., 2006), and in southern Africa (Herremans, 1999; Zhao et al., 2020), where SOI reflects these climate features (McPhaden, Santoso & Cai, 2020). But *Ph. t. trochilus* winters mainly in western Africa (Bensch et al., 2009; Zhao et al., 2020), where TSAH, PSAH and winter NAO reflect climatic conditions (Hurrell, 1995; Munemoto & Tachibana, 2012). Indices of climate in southern and eastern Africa dominated the results of our analysis of the early part of spring; those related to climate in western Africa had a weaker effect on the early passage, but peaked in middle and late spring (Table 3, Fig. 4, Table S14). One possible explanation would be that populations from southern and eastern Africa, probably of *Ph. t. acredula*, begin arriving at Bukowo early in the migration period, and that populations from western Africa, likely of *Ph. t. trochilus*, arrive throughout the migration period, but peak in the middle and last part. This would be a different sequence to that in which they arrive in central Sweden, where *Ph. t. acredula* occur about two weeks later than *Ph. t. trochilus* (Hedenström & Pettersson, 1984; Bensch, Andersson & Åkesson, 1999). Another explanation might be that some *Ph. t. trochilus* during wintering or stopovers visit areas under climatic influence of IOD and SOI, and some *Ph. t. acredula* visit areas influenced by TSAH, PSAH and NAO, for the exact contact zone between the subspecies in Africa remains unknown (Urban, Fry & Keith, 1997; Zhao et al., 2020).

Under the first scenario, *Ph. t. acredula* would arrive early at Bukowo probably because they are heading to their breeding grounds farther north than *Ph. t. trochilus*. Bukowo lies within the southern part of the breeding range of the *trochilus* subspecies, whose northern limits cross central Sweden ca 600–950 km further north (Fig. 1). *Ph. t. acredula* still have to fly another 600–3,000 km from Bukowo to reach their more northerly breeding grounds (Fig. 1). Assuming Willow Warblers’ migrate at 66 km/day in spring, based on ringing recoveries within the Baltic region (Supplement 3), the birds would take about 9 days to cover the 600 km from Bukowo to central Sweden across the Baltic Sea, but several days more to reach northern Sweden, and about a month to reach the northeastern part of their breeding range, where *Ph. t. acredula* predominate (Fig. 1). Under the second scenario, the early cohorts of migrants would mainly consist of populations arriving from the eastern and southeastern part of the species’ wintering range, influenced by the IOD and SOI, but no apparent sequence of subspecies’ arrivals should occur at Bukowo. The sequence of the subspecies’ passage should be verified in future by genotyping (Zhao et al., 2020) and through tracking Willow Warblers by geolocators or other devices (Lerche-Jørgensen et al., 2017; Sokolovskis et al., 2018).

Interannual variations in sex ratios would likely contribute to the effects of climate variables on spring phenology, given Willow Warblers’ protandry (Hedlund et al., 2015) and the likely stronger response of the males to temperatures at the wintering grounds, as in other passerines (Redlisiak, Remisiewicz & Mazur, 2021). Arrivals in spring would therefore be late on average in years with a low proportion of males in the migratory population, despite favourable conditions, and *vice versa*. This could be tested by studies involving DNA sexing (Šoštarić, 2016). Analogously, the year-to-year changes in the proportion of age groups among migrating Willow Warblers’ might also contribute to the effects of climate variables on arrivals at Bukowo. If adults migrate earlier than immatures, as in other passerines (Newton, 2011), then in years with a low proportion of adults in the migratory population of Willow Warblers would arrive later on average in spring than in years with a high proportion of adults. We discuss this possible influence of age ratios in more detail for the SCAND index, but this factor would likely affect all the climate indices.

### Effects of IOD and SOI

The timing of Willow Warblers’ passage through Bukowo in the first main period of spring (1–26 April) was more strongly related to IOD and SOI in Aug–Oct of the previous year than to any other climate index (Table 3, Figs. 4A and 4B, Table S14). The possible explanation might be that the populations visiting eastern and southern parts of the species’ non-breeding range and influenced by these climate indices predominated the passage throughout April, but in May their proportion decreased. The dates when the first 5% of Willow Warblers arrive at the Hanko Bird Observatory in Finland are correlated with April temperatures in the Balkans, southeast of both Hanko and Bukowo (Halkka, Lehikoinen & Velmala, 2011); which is in line with our results. Conditions at eastern African stopover sites, where IOD operates (Fig. 1), influence the birds’ speed at subsequent stages of migration and thus the timing of their arrival in northern Europe, as with Nightingales *Luscinia megarhynchos* and Redstarts *Phoenicurus phoenicurus* (Tøttrup et al., 2012). Willow Warblers on passage to and from southern Africa likely use stopovers in eastern Africa (Fig. 1), which might contribute to the effect IOD shows on the passage at Bukowo. The timing of Willow Warblers’ spring passage was related to IOD and PSAH, as for White Storks *Ciconia ciconia* arriving in Poland from wintering grounds in southeastern and western Africa (Tobolka et al., 2018).

### Effects of precipitation and temperature in the Sahel

The influence of rainfall and temperatures in the Sahel on Willow Warblers’ spring arrivals at Bukowo (Table 3, Figs. 1C–1E) that we found was in line with the influence of these climate features in western Africa on the species’ spring phenology at Helgoland, Germany (Haest, Hüppop & Bairlein, 2020). Those authors interpret this phenomenon as the influence of conditions at wintering or stopover sites in western Africa on populations arriving at Helgoland; we suggest an analogous explanation for Bukowo. Our results, which suggest that Willow Warblers arrive at Bukowo early after a wet and warm August–October of the previous year and after a wet November–December in the Sahel, correspond with the earlier arrival of the species at Helgoland after wet winters in that region of Africa (Haest, Hüppop & Bairlein, 2020). Our results also correspond with the higher survival of Sahara crossings by Willow Warblers after winters with a high Sahel Precipitation Index (Zwarts et al., 2009). The early arrival of spring migrants in Capri, Italy, (Saino et al., 2007) and the Iberian Penninsula (Gordo & Sanz, 2008) was related to wet winters in the Sahel, as in our study. The effect of temperatures in the Sahel that we found corresponds with the early arrival of long-distance migrants, including our study species, in the UK, over 1966–2011 in years with high temperatures in sub-Saharan western and eastern Africa (Cotton, 2003).

### Effects of winter NAO

Many passerines, including Willow Warblers, arrive in Europe early after a positive winter NAO (Dec–March), which is related to an early and warm spring in Europe (*e.g.*, Forchhammer, Post & Stenseth, 2002; Hüppop & Hüppop, 2003; Ahola et al., 2004; Vähätalo et al., 2004; Rainio et al., 2006; Gordo, 2007; Jonzén et al., 2007; Saino & Ambrosini, 2008). The relationship of Willow Warblers’ passage throughout spring at Bukowo to NAO in Nov–March (Table 3, Fig. 1F) corresponds with the relationship between NAO (Dec–March) and all phases of Willow Warbler spring migration through Hanko and Jurmo, Finland, and Helgoland, Germany (Vähätalo et al., 2004; Sparks et al., 2005). NAO (Nov–March) influences the conditions that Willow Warblers experience on spring stopovers in western Africa and in southwestern Europe (Fig. 1). This climate index influenced the species’ timing in all nine sub-periods of spring (Fig. 4F), suggesting that Willow Warblers from these areas migrate through Bukowo throughout the season. The weaker relationship of spring migration timing at Bukowo with NAO (Nov–March) than with other climate indices (Table 3, Fig. 1F) corresponds with winter NAO explaining a small part of the variation in this species’ spring phenology at Helgoland (Haest, Hüppop & Bairlein, 2018). Those results and our study show that other climate indices likely have a greater influence on the spring phenology of this species in Europe than winter NAO.

### Effect of spring NAO

The relationship between Willow Warblers’ migration timing and NAO (Apr–May) that we found (Table 3, Fig. 4G) corresponds with the influence that winds and temperatures in these months at stopovers in southwestern and western Europe have on this species’ spring migration at Helgoland (Haest, Hüppop & Bairlein, 2020). NAO of Apr–May was not related to local temperatures in Łeba in Apr–May (Table S5). Thus the relation of NAO (Apr–May) to Willow Warblers’ passage probably reflected conditions that influnce these birds passage at more distant stopovers than the Polish coast.

### Effects of local temperatures in April–May

We found that local temperatures had no significant effect on the timing of Willow Warblers’ arrivals at Bukowo in the first main period of spring (Table 3, Fig. 4H). This is contrary to correlations found between First Arrival Dates (FADs) of Willow Warblers and other passerines and local spring temperatures at observatories in Helgoland in Germany, Jurmo in Finland, and Rybachy and Ladoga in Russia (Sparks et al., 2005), as well as the relationships between local temperatures and the timing of the first 5% of spring passage at Helgoland, Christiansø in Denmark, and Jurmo (Tøttrup et al., 2010). One reason for these differences might be that FADs are influenced by population numbers, the detectability of the species and observation effort (Tryjanowski & Sparks, 2001; Lindén, 2011). The Annual Anomalies we used are based on a daily percentage of the total catch in a season, and thus are less sensitive to the occurrence of the first birds in an area than studies based on FADs. Another reason would be that we analysed the effect of local temperatures in the context of a far wider range of climate variables, such as SOI and IOD, which seemingly better explained variations in the timing of our study species’ arrivals in early spring. Spring conditions at the Baltic coast, even the most favourable, would not have attracted the migrants any earlier had they not departed early from their wintering grounds several weeks before arrival.

Local temperatures had the greatest effect on Willow Warblers at Bukowo in the middle of spring (Table 3, Fig. 4H), the period where the mean and median dates of the migration passage occur (Remisiewicz & Underhill, 2020). This finding corresponds with correlations of mean or middle dates of this species’ passage with local spring temperatures at other locations in Europe (Sokolov et al., 1998; Hüppop & Hüppop, 2003; Sparks et al., 2005; Lehikoinen et al., 2019). The moderate influence of local temperatures on migration in late spring at Bukowo was analogous to the weak relationships of the 95% of passage in this and other passerines to temperatures at many sites in the northern hemisphere (Lehikoinen et al., 2019).

The pattern of relationships between local temperatures and Willow Warblers’ spring migration timing at Bukowo is likely a combination of several effects. Firstly, birds using a stopover location, such as Bukowo or other bird observatories, do not experience the temperature at that site during the earlier stages of their migration. Any link between their passage and local temperatures is not a cause-and-effect relationship, but the effect of temperatures being correlated over wide areas (Koenig, 1999). The correlation of temperatures in northern Europe with those at previous stopovers in southern Europe and northern Africa might cue long-distance migrants to adjust their arrivals to conditions near and at the breeding grounds (Saino & Ambrosini, 2008; Tøttrup et al., 2008; Tøttrup et al., 2010; Haest, Hüppop & Bairlein, 2020; Brown et al., 2021). Temperatures in southern Europe during stopovers influence the timing of migrants’ arrivals at more northern sites (Gordo, 2007; Halkka, Lehikoinen & Velmala, 2011; Briedis, Hahn & Adamík, 2017).

The second and more direct way that local temperatures influence migration dynamics might be their effect on the availability of insects at stopovers, including those near bird observatories. An abundance of insects depends on weather, which shapes the activity of insectivorous migrants that forage more intensively on warm days than on cold days (Stokke et al., 2005). Intensive foraging by birds ensures good catches in mist nets. The calls of active birds also attract more passing migrants to land at a good stopover site (Chernetsov, 2012). Large catches on days with favourable conditions influence the measures of migration at a site (Knudsen et al., 2007), such as the mean migration dates, dates of percentiles and our Annual Anomaly (Sokolov et al., 1998; Hüppop & Hüppop, 2003; Sparks et al., 2005; Lehikoinen et al., 2019; Remisiewicz & Underhill, 2020). Warm days occur less frequently at Bukowo in the first half of April than later in the season (Šoštarić, 2016), thus the effect of local conditions is likely to be pronounced later in spring, concurring with our results.

### Effects of Scandinavian conditions in summer and the number of juveniles in autumn on the timing of migration in the next spring

The relationship between Willow Warblers’ spring passage through Bukowo and the count of migrating immatures caught the previous August–October and the SCAND index in June–July of the previous year probably reflects conditions in the breeding season preceding spring passage. Spring migration at Bukowo shifted later when SCAND (Jun–July_1y) was low and the count of juveniles in the previous autumn was large (Njuv_1y), and *vice versa* (Table 3, Fig. 5). The effect of SCAND (Jun–July_1y) on the mid- and last three main periods of spring passage (Table 3, Fig. 4I) was the strongest shown by all the climate variables, in line with the conclusion of Ockendon, Leech & Pearce-Higgins (2013) that conditions on the breeding grounds are the main drivers of changes in the breeding phenology and nesting success for long-distance migrants, including Willow Warblers. The partial AAs reflect the timing and the numbers of the migration passage through Bukowo in each period of spring in relation to a multiyear baseline (Remisiewicz & Underhill, 2020). Thus AA might relate not only to climatic variation that influences the timing of arrivals but also to the demography of the spring passage (Gordo, 2007).

Unfavourable weather during breeding limits the availability of insects, thus also the survival of insectivores and their nestlings, and so the demography of a population (Stokke et al., 2005). In summer, the positive phase of SCAND is related to high temperatures and low precipitation in Scandinavia (Bueh & Nakamura, 2007), which should provide good feeding conditions for the insectivorous Willow Warblers in the breeding season (Fig. 1). Occurrences of Willow Warblers in greater numbers at Bukowo in springs after summers with a high SCAND, and thus likely a more successful previous breeding season in Scandinavia, would shift the bulk of the species’ passage to later dates, as the cohorts that responded to SCAND occurred at Bukowo in middle and late periods of spring. This would cause negative values of the AA index for late passage (Remisiewicz & Underhill, 2020).

Nevertheless, the lack of a correlation between the count of juvenile Willow Warblers in autumn at Bukowo and SCAND in June–July the previous year suggested that summer conditions in Scandinavia were not the main underlying reason for the relationship between Njuv_1y in autumn the previous year and the timing of migration in spring. The lack of a relationship might be an effect of the sequential passage of different migratory populations in autumn and in spring, including *Ph. t. acredula* from breeding grounds east of the influence of SCAND (Fig. 1). Based on ringing recoveries from Willow Warblers’ breeding grounds north of Bukowo (Fransson & Hall-Karlsson, 2008; Valkama et al., 2014; Maciąg et al., 2017), we had assumed that the same breeding populations migrate through the site in spring and autumn, but likely in different proportions over the seasons and the years, depending on local weather. Juveniles constituted 76–100% of all Willow Warblers caught in autumn (Table S2), probably because of the “coastal effect”, whereby young birds use stopovers on the coastline more often than the more experienced adults (Payevsky, 1998). Thus we did not use the proportion of juveniles to adults as a measure of the species’ breeding success, as in other studies (*e.g.*, Barshep et al., 2011; Newton & Dawson, 2011).

Young Willow Warblers at the end of their first year of life are indistinguishable from adults (Svensson, 1992; Demongin, 2016) after a complete moult at the wintering grounds (Underhill et al., 1992), so we could not age the birds we caught in spring at Bukowo. But if adults arrive at the breeding grounds ahead of young birds, as in other passerines (Newton, 2011), then adults likely dominated the cohort of early migrants of this species and the young birds arrived later at Bukowo on average. Young birds would be better represented after a summer with high breeding success more than after a poor breeding season. Young birds arriving in spring later than adults, and in large numbers after summers with good breeding success, would shift the peak of passage later, reflected by lower AA values in mid-spring, than after summers with low breeding success. Thus we suggest that the later spring migration at Bukowo after a higher count of Njuv in autumn probably reflected the demography of migrating cohorts of Willow Warblers.

## Conclusions

We showed that the pattern of Willow Warbler arrivals in spring at the southern coast of the Baltic was sequentially influenced by indices of climate in different parts of the species’ winter range. The climatic variability that some populations experienced on their wintering grounds in southern and eastern Africa had carryover effects on the timing of the species’ arrivals in northern Europe in early spring; the indices of climate in western Africa strongly affected the populations arriving in the middle and the end of spring. Climate change and year-to-year variation of weather at the wintering grounds enable long-distance migrants to depart earlier after favourable conditions (*e.g.*, Katti & Price, 1999; González, Bayly & Hobson, 2021; Tomotani et al., 2021). Conditions at stopover sites influence the rate of spring migration (*e.g.*, Tøttrup et al., 2012; Aloni, Markman & Ziv, 2019). The gradually warmer spring season in the northern hemisphere favours the early breeding of migrants (Ouwehand & Both, 2017; Hoover & Schelsky, 2020). Our results showed that climate changes at remote wintering grounds and at stopover sites are important drivers of changes in the arrival timing of migrants in Europe. These factors operate in combination with climate change in the northern hemisphere. The overall effect is a shift to earlier spring arrivals in the north that enables earlier breeding, which has been shown to contribute to phenological shifts in the life stages of long-distance migrants such as Barn Swallow (Burman, 2016), Pied Flycatcher (Ouwehand & Both, 2017; Tomotani et al., 2018) and Willow Warbler (Hedlund et al., 2015; Remisiewicz & Underhill, 2020; this study). We propose that the analysis of temporal changes in the relationships between migration parameters and climate indices at different winter quarters and at stopovers is a useful tool to unravel the arrival patterns in species that use widespread wintering grounds, such as many Euro-African migrant birds.

## Supplemental Information

10.7717/peerj.12964/supp-1 Supplemental Information 1 Supplementary Figures and Tables.

10.7717/peerj.12964/supp-2 Supplemental Information 2 Ringing data Willow Warblers ringed at Bukowo in springs 1982–2017, and in autumns 1981–2017, used in the study.

10.7717/peerj.12964/supp-3 Supplemental Information 3 Direct ringing recoveries of Willow Warblers ringed or recovered in spring at Bukowo in 1982-2017 used in the study. Direct short-term ringing recoveries of Willow Warblers within spring migration, which were used to calculate mean speed of spring migration.

10.7717/peerj.12964/supp-4 Supplemental Information 4 RScript and data files used in the study.

## Additional Information and Declarations

### Field Study Permissions

The following information was supplied relating to field study approvals (*i.e*., approving body and any reference numbers): Permit to conduct field research at Bukowo from the Marine Office, Słupsk (OW-A-510/87/17/ds).

### Data Availability

The following information was supplied regarding data availability: The raw data are available in the Supplemental Files.
