# Supplementary material for: Climate in Africa sequentially shapes spring passage of Willow Warbler Phylloscopus trochilus across the Baltic coast
Source: PeerJ. 2022 Feb 18;10:e12964. doi: 10.7717/peerj.12964 (PMC8860065; doi:10.7717/peerj.12964)
Supplement: Supplemental Information 1 [file peerj-10-12964-s001.pdf]

Climate in Africa sequentially shapes spring passage of Willow Warbler *Phylloscopus trochilus* across the Baltic coast

Supplementary Information

Figures S1–S5 and Tables S1–S16

Magdalena Remisiewicz[1,2], Les G. Underhill[2,3]

[1] Bird Migration Research Station, Faculty of Biology, University of Gdańsk, Wita Stwosza 59, 80-308, Gdańsk, Poland; https://orcid.org/0000-0002-3613-5738;
Email address: magdalena.remisiewicz@ug.edu.pl
[2] Department of Biological Sciences, University of Cape Town, Rondebosch, 7701, South Africa; https://orcid.org/0000-0002-8758-1527
[3] Biodiversity and Development Institute, 25 Old Farm Road, Rondebosch, 7700, Cape Town 7475, South Africa
Email address: les.underhill@uct.ac.za

Corresponding Author:
Magdalena Remisiewicz
Email address: magdalena.remisiewicz@ug.edu.pl

Figure S1. Cumulative count of Willow Warblers for each spring in 1982–2017 and the multiyear average cumulative count. The area between the two curves reflects the overall Annual Anomaly for a season (see Remisiewicz & Underhill, 2020). The vertical lines divide each AA into three main periods: MP1 (1–26 April), MP2 (27 April–5 May), MP3 (6–15 May), which sum up to overall AA.

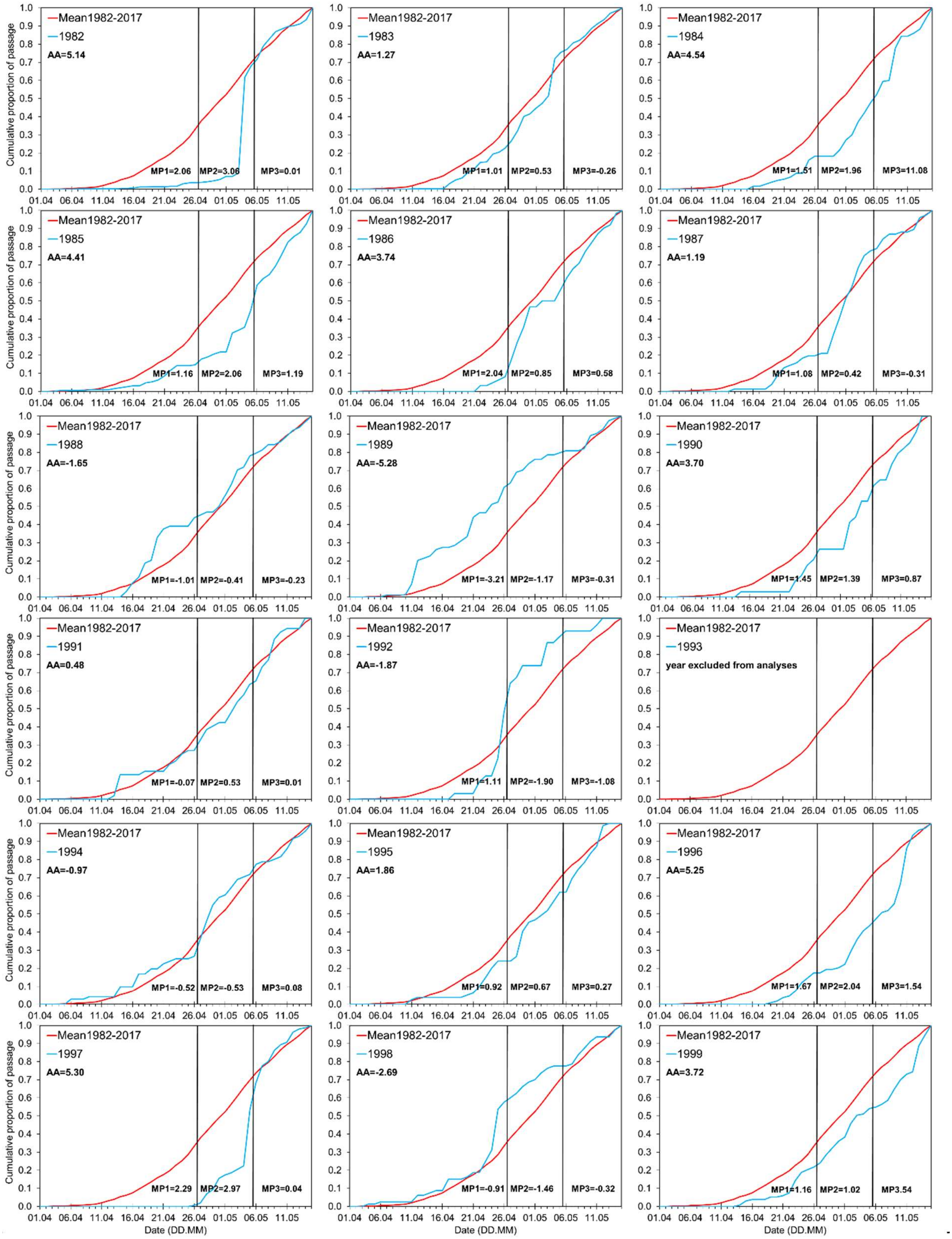

Figure S1 continued.

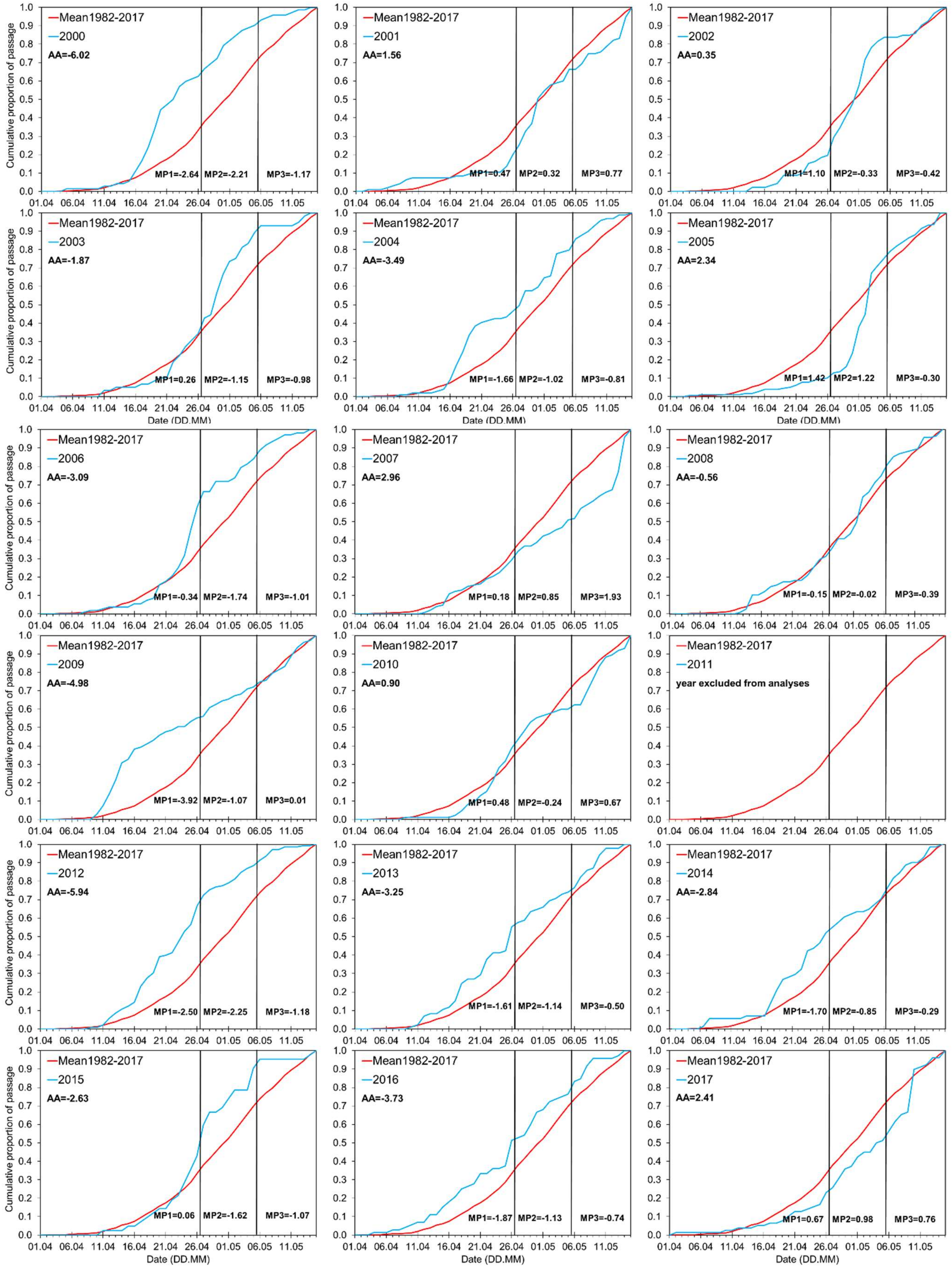

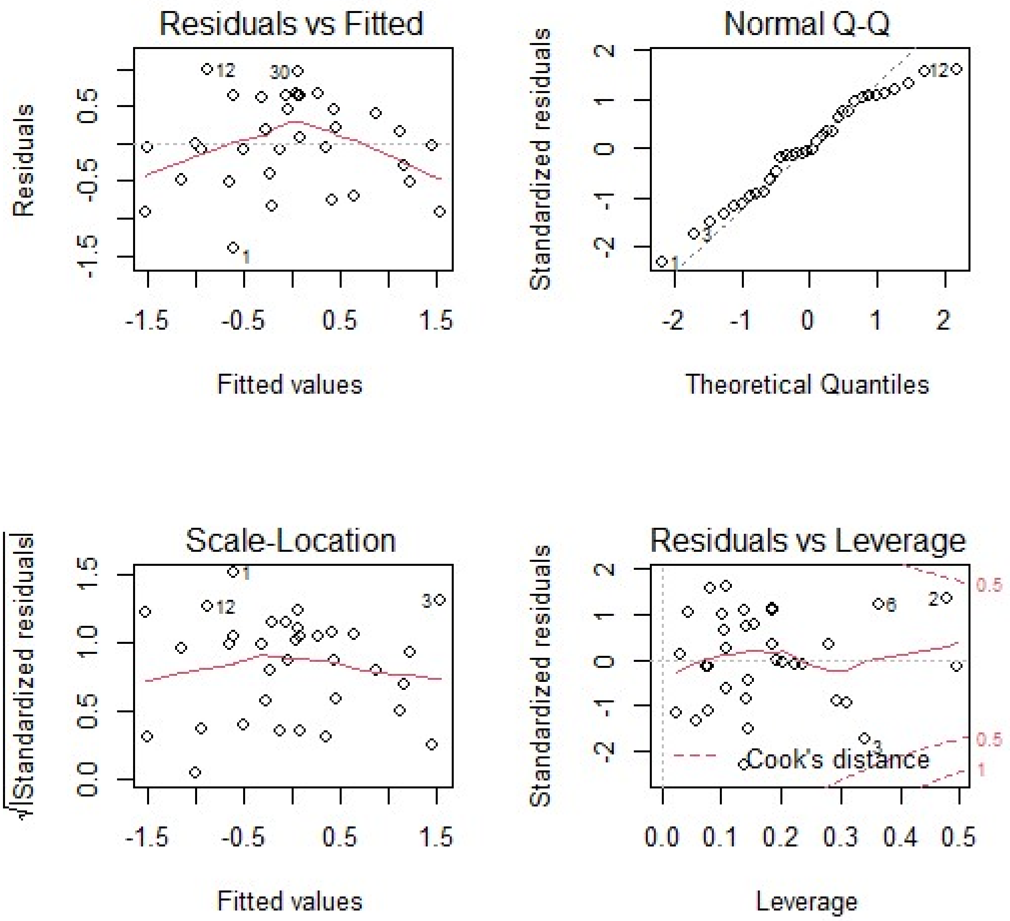

Figure S2. Model diagnostics for the best model with MP1 as the explained variable, and 15 climate variables and the Year as the explanatory variables (Table 3). The plots of residuals follow Crawley (2013).

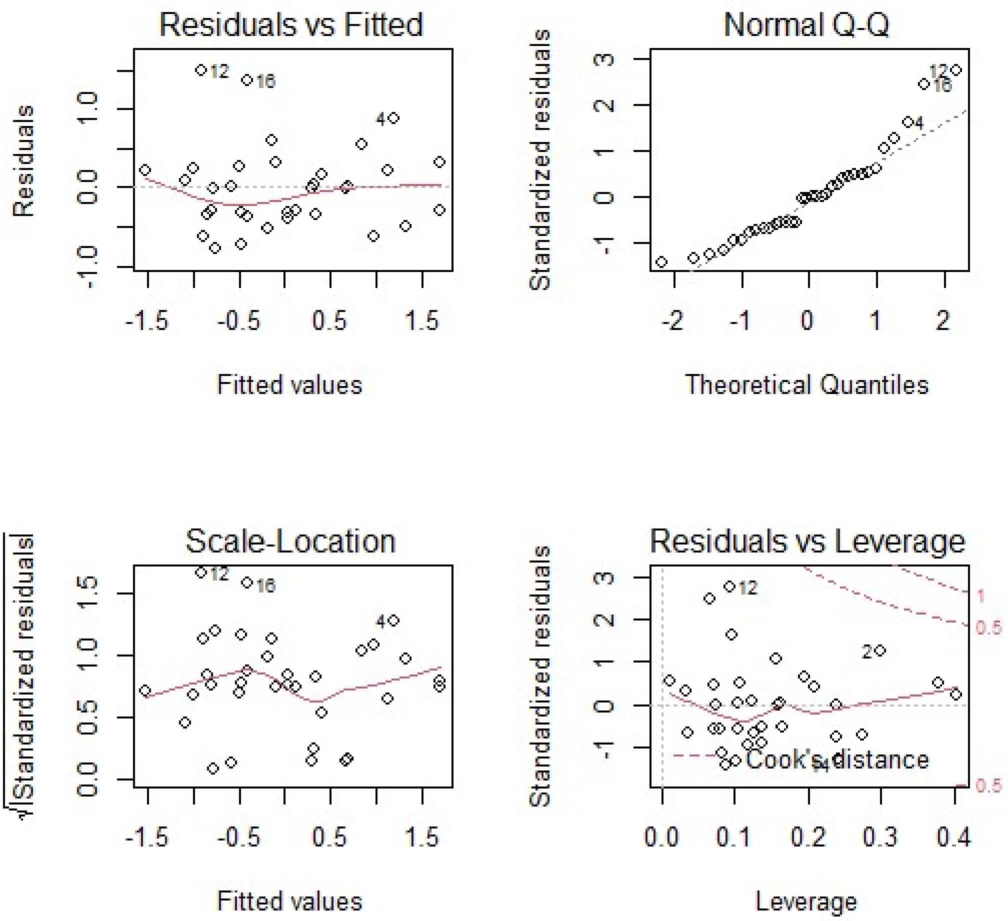

Figure S3. Model diagnostics for the best model with MP2 as the explained variable, and 15 climate variables and the Year as explanatory variables (Table 3). The plots of residuals follow Crawley (2013).

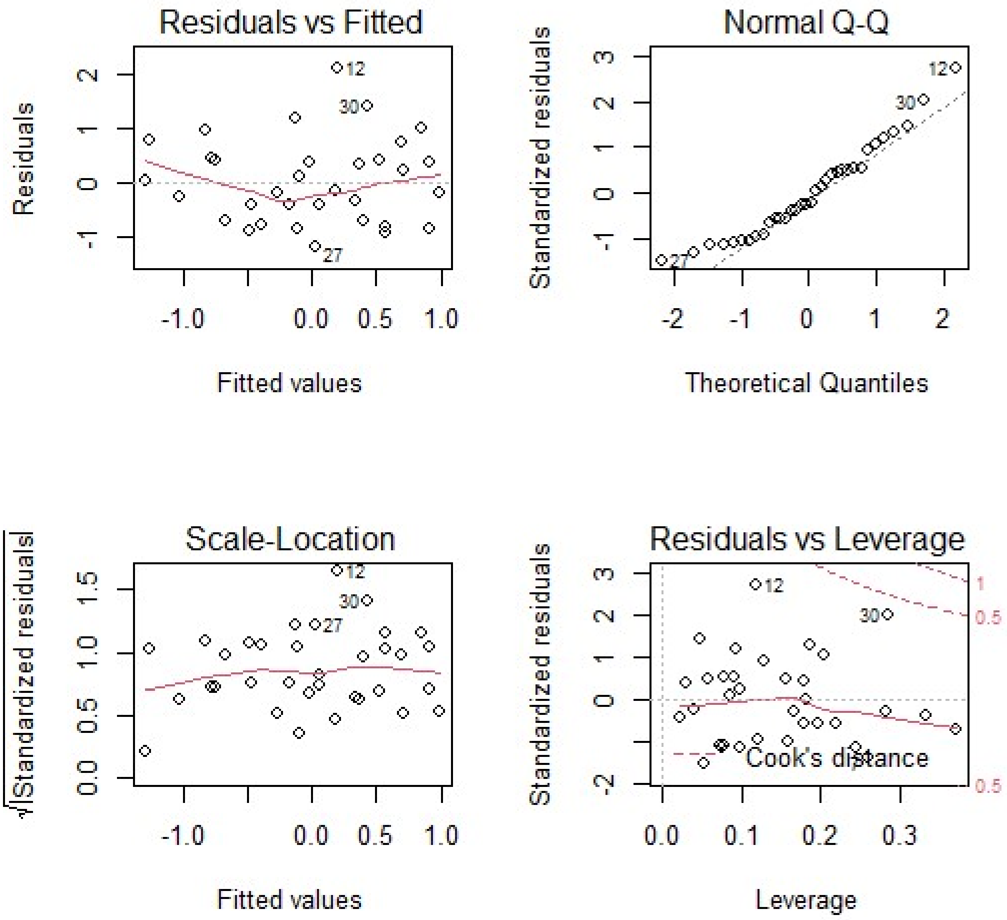

Figure S4. Model diagnostics for the best model with MP3 as the explained variable, and 15 climate variables and the Year as explanatory variables (Table 3). The plots of residuals follow Crawley (2013).

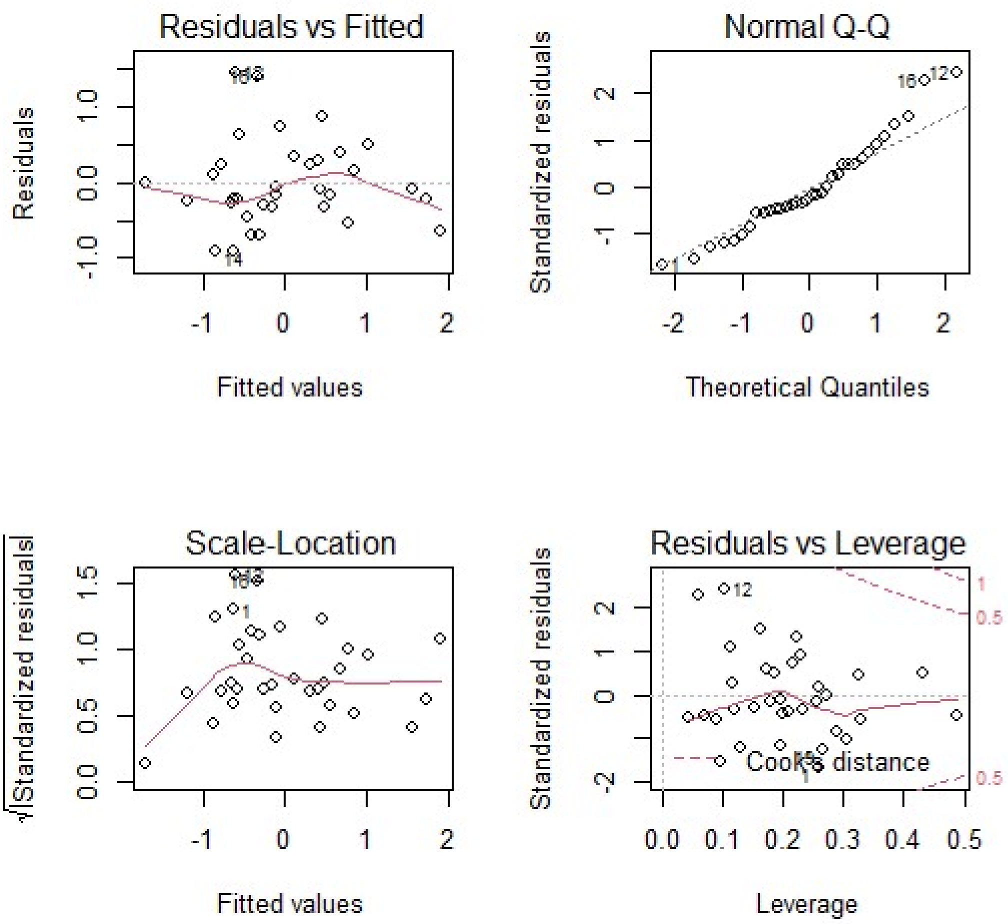

Figure S5. Model diagnostics for the best model with overall spring AA as the explained variable, and 15 climate variables and the Year as explanatory variables (Table 3). The plots of residuals follow Crawley (2013).

Table S1. The numbers of Willow Warblers ringed each spring and autumn in 1981–2017 at both locations of the Bukowo ringing station, the numbers of mist nets used and numbers of birds caught each season of the study. The two locations are 16 km apart: Kopań (54°27'11"N, 16°24'08"E) and Bukowo (54°20'13"N, 16°14'36"E). N = numbers of Willow Warblers caught each spring (1 April–15 May), aged as "full grown"; N nets = number of 8 m passerine mist nets used each season, N juv = total number of juvenile Willow Warblers caught each autumn, N juvAB_50 = number of juveniles caught each autumn recalculated as catch per 50 mist nets. "–" = seasons excluded from this study because fewer than 30 birds were caught.

		Spring		Autumn		
Year	Location	N	N nets	N juv	N nets	N juvAB_50
1981	Kopań	–	57	689	45	765
1982	Kopań	1016	57	1048	50	1048
1983	Kopań	498	57	440	53	415
1984	Kopań	180	42	119	38	156
1985	Kopań	189	51	152	44	173
1986	Kopań	62	50	67	43	78
1987	Kopań	76	45	40	55	36
1988	Kopań	64	45	39	55	35
1989	Kopań	84	45	46	45	51
1990	Kopań	34	45	205	45	228
1991	Kopań	52	45	40	46	43
1992	Kopań	31	37	100	49	102
1993	Kopań	–	47	286	55	260
1994	Kopań	71	47	132	47	140
1995	Kopań	79	47	257	55	234
1996	Kopań	108	47	444	70	317
1997	Kopań	161	47	151	60	126
1998	Kopań	80	47	643	64	502
1999	Kopań	133	47	176	60	147
2000	Kopań	72	47	228	60	190
2001	Kopań	95	57	169	60	141
2002	Kopań	92	47	303	65	233
2003	Kopań	58	46	175	69	127
2004	Kopań	99	47	223	70	159
2005	Kopań	177	47	119	76	78
2006	Kopań	107	47	182	60	152
2007	Kopań	147	54	169	62	136
2008	Kopań	115	50	89	62	72
2009	Kopań	107	46	125	64	98
2010	Kopań	85	35	62	63	49
2011	–	–	–	–	–	–
2012	Bukowo	138	56	50	56	45
2013	Bukowo	85	51	87	42	104
2014	Bukowo	71	48	130	48	135
2015	Bukowo	42	51	110	51	108
2016	Bukowo	72	52	64	48	63
2017	Bukowo	78	52	60	54	62

Table S2. Counts of Willow Warblers ringed each spring and autumn in 1981–2017 at Bukowo, Baltic Sea coast, Poland. N = numbers of Willow Warblers aged as "full grown" during spring migration (1 April–15 May) used to calculate the Annual Anomaly for the whole spring season (AA) and for the three main periods of spring passage (MP1, MP2, MP3). N juvAB_50 = numbers of juvenile Willow Warblers caught at Bukowo during autumn migration (14 August–29 October) in 1981–2018, recalculated as catches per 50 mist nets (as in Table S1), % juv aut = proportion of juveniles among Willow Warblers caught at Bukowo during autumn migration; "–" = seasons excluded from analyses because fewer than 30 Willow Warblers were caught. Symbols and dates as in Table 2 and Fig. S1.

Year	N MP1	N MP2	N MP3	N AA	AA MP1	AA MP2	AA MP3	AA	NjuvAB _50	% juv aut
1981	–	–	–	–	–	–	–	–	765	94%
1982	36	592	388	1016	2.14	3.15	0.02	5.14	1048	82%
1983	102	256	140	498	1.08	0.55	–0.18	1.27	415	84%
1984	29	47	104	180	1.58	2.25	0.89	4.54	156	94%
1985	27	40	122	189	1.24	2.39	0.96	4.41	173	88%
1986	4	27	31	62	2.12	1.06	0.47	3.47	78	100%
1987	15	42	19	76	1.15	0.42	–0.20	1.19	36	100%
1988	25	21	18	64	–0.94	–0.42	–0.12	–1.65	35	94%
1989	44	22	18	84	–3.14	–1.79	–0.18	–5.28	51	98%
1990	6	12	16	34	1.52	1.63	0.73	3.70	228	100%
1991	14	16	22	52	0.01	0.67	–0.03	0.48	43	94%
1992	7	20	4	31	1.19	–2.03	–0.85	–1.87	102	92%
1993	–	–	–	–	–	–	–	–	260	87%
1994	18	32	21	71	–0.44	–0.47	0.12	–0.97	140	89%
1995	19	26	34	79	1.00	0.83	0.21	1.86	234	83%
1996	16	28	64	108	1.75	2.37	1.30	5.25	317	87%
1997	0	36	125	161	2.37	3.21	–0.10	5.30	126	97%
1998	43	19	18	80	–0.84	–1.46	–0.22	–2.69	502	90%
1999	27	41	65	133	1.23	1.26	1.40	3.72	147	87%
2000	44	20	8	72	–2.57	–2.34	–0.94	–6.02	190	98%
2001	13	44	38	95	0.54	0.43	0.76	1.56	141	91%
2002	17	58	17	92	1.18	–0.40	–0.25	0.35	233	87%
2003	18	31	10	58	0.34	–1.27	–0.76	–1.87	127	76%
2004	43	35	21	99	–1.58	–1.04	–0.69	–3.49	159	96%
2005	17	109	51	177	1.50	1.24	–0.22	2.34	78	90%
2006	49	38	20	107	–0.27	–1.81	–0.84	–3.09	152	95%
2007	38	31	78	147	0.26	1.11	1.76	2.96	136	81%
2008	34	48	33	115	–0.08	0.00	–0.31	–0.56	72	94%
2009	57	19	31	107	–3.85	–1.01	0.06	–4.98	98	95%
2010	27	24	34	85	0.55	–0.06	0.59	0.90	49	96%
2011	–	–	–	–	–	–	–	–	–	–
2012	78	42	18	138	–2.43	–2.36	–0.97	–5.94	45	93%
2013	36	26	23	85	–1.53	–1.11	–0.43	–3.25	104	93%
2014	33	15	23	71	–1.63	–0.78	–0.26	–2.84	135	97%
2015	15	18	9	42	0.14	–1.75	–0.84	–2.63	108	94%
2016	27	27	18	72	–1.79	–1.12	–0.64	–3.73	63	82%
2017	13	26	39	78	0.75	1.24	0.60	2.41	62	84%

Table S3. Comparison of numbers of Willow Warblers caught at Kopań and Bukowo. NAA = numbers of Willow Warblers caught in spring used to calculate the Annual Anomaly for 1982–2017; N/net spring = number of birds caught per net each spring; N juvAB_50 = numbers of juvenile Willow Warblers caught at Bukowo during autumn migration in 1981–2018, recalculated per 50 mist nets; Njuv/net autumn = number of juveniles caught per net each autumn. Data for this comparison is in Tables S1 and S2.

	N years		U-test	
Variable	Kopań	Bukowo	U	p
**Spring**				
NAA	28	6	58.5	0.259
N/net spring	28	6	46.5	0.095
**Autumn**				
N juvAB_50	30	6	45.0	0.059
Njuv/net autumn	30	6	45.0	0.059

Table S4. Relationships between the numbers of caught birds (NAA) and the Year (YearN), and the Annual Anomaly in the timing of migration for three main periods of spring (MP1, MP2, MP3) and the whole spring (AA) for Willow Warblers caught at Bukowo, Poland, in 1982–2017. Estimate – coefficients from multiple regression, SE – standard error of the estimates; *t, p* – *t*-test and significance of each estimate, $p < 0.05$ marked in **bold** face.

Explanatory variable	Estimate	SE	*t*	*p*
**MP1**	Model statistics: $F_{2,31} = 4.21$, $AdjR^2 = 16.3\%$			
YearN	**-0.06**	**0.03**	**-2.26**	**0.031**
N AA	0.00	0.00	0.82	0.416
Intercept	0.98	0.64	1.53	0.136
**MP2**	Model statistics: $F_{2,31} = 6.21$, $AdjR^2 = 24.0\%$			
YearN	-0.05	0.02	-2.03	0.051
N AA	0.00	0.00	1.90	0.066
Intercept	0.61	0.60	1.01	0.322
**MP3**	Model statistics: $F_{2,31} = 1.05$, $AdjR^2 = 0.3\%$			
YearN	-0.02	0.01	-1.40	0.171
N AA	0.00	0.00	-0.19	0.852
Intercept	0.37	0.31	1.18	0.245
**AA**	Model statistics: $F_{2,31} = 5.11$, $AdjR^2 = 19.9\%$			
YearN	**-0.13**	0.05	-2.29	**0.029**
N AA	0.00	0.00	1.19	0.242
Intercept	1.78	1.36	1.31	0.199

Table S5. Pearson's correlation coefficients of climate indices in 1981–2017 used in multiple regression models. Abbreviations of the climate variables as in Table 1; "_1y" = climate indices from the year preceding the spring being analysed. Significant correlations after Benjamini-Hochberg correction for multiple comparisons with accepted 5% level of false positives marked in **bold** face.

Variable	TLEB APR_MAY	NAO APR_MAY	SCAND APR_MAY	NAO NOV _MAR	PSAH NOV _MAR	TSAH NOV _MAR	IOD NOV _MAR	SOI NOV _MAR	NAO AUG _OCT_1y	SAH AUG _OCT_1y	TSAH AUG _OCT_1y	IOD AUG _OCT_1y	SOI AUG _OCT_1y	NAO JUN _JUL_1y
TLEB_APR_MAY														
NAO_APR_MAY	-0.09													
SCAND_APR_MAY	-0.08	-0.02												
NAO_NOV_MAR	0.02	0.17	-0.10											
PSAH_NOV_MAR	0.07	-0.01	-0.01	0.07										
TSAH_NOV_MAR	0.28	-0.22	0.03	-0.53	0.24									
IOD_NOV_MAR	0.06	-0.27	-0.01	-0.01	-0.10	0.30								
SOI_NOV_MAR	-0.02	-0.02	-0.20	-0.06	0.05	-0.09	-0.11							
NAO_AUG_OCT_1y	-0.31	-0.04	0.27	-0.12	0.00	-0.01	-0.37	-0.09						
SAH_AUG_OCT_1y	0.28	0.02	-0.15	0.27	0.11	0.08	0.39	0.11	-0.29					
TSAH_AUG_OCT_1y	0.29	-0.25	-0.02	-0.24	0.02	0.69	0.22	-0.14	-0.14	-0.28				
IOD_AUG_OCT_1y	0.07	-0.18	0.27	0.19	-0.08	0.09	**0.60**	-0.40	-0.21	0.24	0.09			
SOI_AUG_OCT_1y	-0.16	0.15	-0.17	-0.23	-0.09	-0.05	-0.33	**0.82**	0.11	-0.02	-0.16	**-0.60**		
NAO_JUN_JUL_1y	0.16	0.14	0.37	-0.11	0.11	-0.10	-0.46	-0.10	0.02	-0.13	-0.22	-0.32	-0.03	
SCAND_JUN_JUL_1y	-0.39	-0.09	0.16	0.00	-0.22	-0.02	0.35	0.06	0.26	-0.16	-0.06	0.23	0.08	-0.38

Table S6. Summary statistics for linear regressions over the year in 1982–2017 for the climate variables used in the study. June–July and August–October means are averages in 1981–2016 for the season preceding Willow Warblers' spring migrations in 1982–2017. Values averaged for the given range of months and used as raw (non-standardised) data. Abbreviations of variables as in Table 1. *P* < 0.05 marked in **bold** face.

Parameter	*ß* slope	SE	R^2	t_{32}	*P*
**TLEB Apr–May**	**0.0406**	0.0132	0.23	3.07	**0.0044**
NAO Apr–May	-0.0099	0.0123	0.02	-0.80	0.4272
SCAN Apr–May	0.0028	0.0104	0.002	0.27	0.7902
NAO Nov–Mar	0.1403	0.0962	0.06	1.46	0.1543
PSAH Nov–Mar	0.0177	0.0165	0.03	1.07	0.2907
**TSAH Nov–Mar**	**0.0349**	0.0073	0.40	4.75	**0.00004**
**IOD Nov–Mar**	**0.0066**	0.0030	0.13	2.22	**0.0338**
SOI Nov–Mar	0.0176	0.0088	0.11	1.99	0.0548
NAO Aug–Oct_1y	-0.0143	0.0090	0.07	-1.60	0.1204
**PSAH Aug–Oct_1y**	**8.4100**	2.4360	0.27	3.45	**0.0016**
**TSAH Aug–Oct_1y**	**0.0167**	0.0053	0.23	3.17	**0.0032**
IOD Aug–Oct_1y	0.0034	0.0134	0.002	0.26	0.8004
SOI Aug–Oct_1y	0.0083	0.0062	0.05	1.35	0.1871
**NAO Jun–Jul**_1y	**-0.0310**	0.0127	0.16	-2.44	**0.0204**
SCAND Jun–Jul_1y	-0.0139	0.0165	0.02	-0.84	0.4065

Table S7. Summary statistics for linear regressions on the metrics of spring migration phenology of Willow Warblers at Bukowo, Poland, in 1982–2017. Plots for the three main periods of spring (MP1, MP2, MP3) and for the overall spring Annual Anomaly (AA) are presented in Fig. 3. *ß* slope = regression coefficient; SE = Standard Error; R^2 – determination coefficient; *t, p* = results of *t*-test, significant p values after applying Benjamini-Hochberg correction (Benjamini & Hochberg, 1995) for multiple comparisons marked in **bold** face, 36 x *ß* = estimated change in days of migration timing over 1982–2017, negative values reflect earlier migration.

Parameter	*ß* slope	SE	R^2	t_{34}	*p*	36 years * *ß* (days)
MP1	–0.07	0.02	0.20	–2.80	**0.009**	–2.42
MP2	–0.06	0.02	0.21	–2.94	**0.006**	–2.29
MP3	–0.02	0.01	0.06	–1.48	0.149	–0.72
AA	–0.15	0.05	0.21	–2.95	**0.006**	–5.43

Table S8. Relationships between 13 climate variables and year in full models, and Annual Anomaly in the timing of migration for three main periods of spring (MP1, MP2, MP3) and whole spring (AA) for Willow Warblers caught at Bukowo, Poland, in 1982–2017. Estimate – coefficients from multiple regression, SE – standard error of the estimates; *t, p* – *t*-test and significance of each estimate, $p < 0.05$ marked in **bold** face, $0.05 < p < 0.1$ in ***bold italics***. VIF – variance inflation factor. R^2 – partial determination coefficients, *pR* – Pearson's partial correlation coefficient. Symbols for climate variables as in Table 1, for periods as in Table 2.

Explanatory variable	Estimate	SE	*t*	*p*	VIF	R^2	*pR*
**MP1** Full model: $F_{16,18} = 2.591$, $AdjR^2 = 42.8\%$							
YearN	0.00	0.01	0.26	0.797	1.29	0.00	0.22
LEB_APR_MAY	-0.08	0.17	-0.49	0.632	1.72	0.01	-0.20
NAO_APR_MAY	-0.35	0.15	-2.32	**0.032**	1.35	0.23	-0.49
SCAND_APR_MAY	-0.05	0.19	-0.28	0.780	2.08	0.00	-0.05
NAO_NOV_MAR	-0.20	0.23	-0.88	0.391	3.03	0.04	-0.25
PSAH_NOV_MAR	-0.11	0.17	-0.67	0.509	1.64	0.02	-0.21
TSAH_NOV_MAR	0.10	0.34	0.28	0.782	6.71	0.00	-0.03
IOD_NOV_MAR	-0.17	0.25	-0.67	0.514	3.83	0.02	-0.08
SOI_NOV_MAR	-0.26	0.29	-0.88	0.390	5.02	0.04	-0.23
NAO_AUG_OCT_1y	-0.13	0.19	-0.70	0.493	2.19	0.03	-0.13
PSAH_AUG_OCT_1y	-0.34	0.26	-1.32	0.202	3.89	0.09	-0.36
TSAH_AUG_OCT_1y	-0.39	0.31	-1.25	0.229	5.84	0.08	-0.33
IOD_AUG_OCT_1y	-0.49	0.24	-2.08	***0.052***	3.34	0.19	-0.47
SOI_AUG_OCT_1y	-0.36	0.35	-1.04	0.311	7.24	0.06	-0.26
NAO_JUN_JUL_1y	-0.04	0.22	-0.17	0.866	2.80	0.00	0.04
SCAND_JUN_JUL_1y	-0.20	0.20	-0.99	0.334	2.37	0.05	-0.28
**MP2** Full model: $F_{16,18} = 4.547$, $AdjR^2 = 62.5\%$							
YearN	0.00	0.01	0.16	0.871	1.29	0.00	0.13
**LEB_APR_MAY**	-0.42	0.14	-3.09	**0.006**	1.72	0.35	-0.58
**NAO_APR_MAY**	-0.51	0.12	-4.19	**0.001**	1.35	0.49	-0.71
SCAND_APR_MAY	0.04	0.15	0.30	0.771	2.08	0.00	0.08
NAO_NOV_MAR	-0.14	0.18	-0.78	0.447	3.03	0.03	-0.21
PSAH_NOV_MAR	-0.14	0.13	-1.01	0.326	1.64	0.05	-0.26
TSAH_NOV_MAR	0.00	0.28	-0.01	0.993	6.71	0.00	-0.05
IOD_NOV_MAR	0.07	0.21	0.32	0.753	3.83	0.01	0.11
SOI_NOV_MAR	-0.10	0.24	-0.41	0.689	5.02	0.01	-0.11
NAO_AUG_OCT_1y	-0.16	0.16	-1.02	0.319	2.19	0.06	-0.21
PSAH_AUG_OCT_1y	-0.31	0.21	-1.49	0.153	3.89	0.11	-0.34
TSAH_AUG_OCT_1y	-0.26	0.25	-1.02	0.323	5.84	0.05	-0.26
**IOD_AUG_OCT_1y**	-0.48	0.19	-2.48	**0.023**	3.34	0.26	-0.52
SOI_AUG_OCT_1y	-0.10	0.28	-0.34	0.736	7.24	0.01	-0.10
NAO_JUN_JUL_1y	-0.07	0.18	-0.41	0.686	2.80	0.01	-0.05
**SCAND_JUN_JUL_1y**	-0.52	0.16	-3.22	**0.005**	2.37	0.36	-0.61

Table S8. continued

Explanatory variable	Estimate	SE	*t*	*p*	VIF	R^2	*pR*
**MP3** Full model: $F_{16,18} = 1.164$, $AdjR^2 = 7.2\%$							
YearN	0.00	0.01	0.07	0.946	1.29	0.00	0.15
LEB_APR_MAY	-0.37	0.22	-1.69	0.108	1.72	0.14	-0.39
NAO_APR_MAY	-0.26	0.19	-1.34	0.198	1.35	0.09	-0.30
SCAND_APR_MAY	0.18	0.24	0.74	0.471	2.08	0.03	0.19
NAO_NOV_MAR	-0.37	0.29	-1.28	0.218	3.03	0.08	-0.31
PSAH_NOV_MAR	-0.34	0.21	-1.61	0.124	1.64	0.13	-0.38
TSAH_NOV_MAR	-0.23	0.44	-0.53	0.601	6.71	0.02	-0.17
IOD_NOV_MAR	0.08	0.32	0.26	0.801	3.83	0.00	0.11
SOI_NOV_MAR	0.19	0.37	0.53	0.605	5.02	0.02	0.10
NAO_AUG_OCT_1y	-0.11	0.25	-0.45	0.655	2.19	0.01	-0.08
PSAH_AUG_OCT_1y	0.11	0.33	0.34	0.737	3.89	0.01	-0.01
TSAH_AUG_OCT_1y	0.17	0.40	0.42	0.682	5.84	0.01	0.05
IOD_AUG_OCT_1y	-0.26	0.30	-0.86	0.401	3.34	0.04	-0.23
SOI_AUG_OCT_1y	-0.16	0.44	-0.37	0.715	7.24	0.01	-0.10
NAO_JUN_JUL_1y	0.11	0.28	0.41	0.685	2.80	0.01	0.14
SCAND_JUN_JUL_1y	-0.32	0.25	-1.26	0.226	2.37	0.08	-0.31
**AA** Full model: $F_{16,18} = 3.066$, $AdjR^2 = 49.3\%$							
YearN	0.00	0.01	0.21	0.840	1.29	0.00	0.20
TLEB_APR_MAY	-0.31	0.16	-1.91	***0.072***	1.72	0.17	-0.45
**NAO_APR_MAY**	-0.44	0.14	-3.10	**0.006**	1.35	0.35	-0.60
SCAND_APR_MAY	0.04	0.18	0.21	0.838	2.08	0.00	0.07
NAO_NOV_MAR	-0.24	0.21	-1.13	0.273	3.03	0.07	-0.30
PSAH_NOV_MAR	-0.19	0.16	-1.23	0.236	1.64	0.08	-0.32
TSAH_NOV_MAR	-0.01	0.32	-0.03	0.974	6.71	0.00	-0.09
IOD_NOV_MAR	-0.03	0.24	-0.13	0.899	3.83	0.00	0.04
SOI_NOV_MAR	-0.11	0.27	-0.41	0.689	5.02	0.01	-0.12
NAO_AUG_OCT_1y	-0.16	0.18	-0.87	0.397	2.19	0.04	-0.17
PSAH_AUG_OCT_1y	-0.26	0.24	-1.08	0.293	3.89	0.06	-0.31
TSAH_AUG_OCT_1y	-0.25	0.29	-0.85	0.405	5.84	0.04	-0.25
**IOD_AUG_OCT_1y**	-0.49	0.22	-2.21	**0.041**	3.34	0.21	-0.49
SOI_AUG_OCT_1y	-0.25	0.33	-0.76	0.456	7.24	0.03	-0.20
NAO_JUN_JUL_1y	-0.02	0.20	-0.10	0.922	2.80	0.00	0.05
SCAND_JUN_JUL_1y	-0.39	0.19	-2.07	***0.053***	2.37	0.19	-0.47

Table S9. Model selection procedure by "all subsets", according to AICc, from the full model (Table S8) of the relationship between the 15 climate variables and the year, and timing of migration in the first main period of spring (MP1) for Willow Warblers caught at Bukowo, Poland, in 1982–2017. The table presents all models with ΔAICc < 2. The models ranked by corrected Akaike's Information Criteria for small samples size (AICc), *k* is the number of estimated parameters in the model, ΔAICc gives the difference in AICc from the model with lowest AICc, w_i is the Akaike weight. The best model, discussed in the text, in **bold** face. Model selection with the package MuMIn 1.43.6 (Bartoń, 2020) in R 4.0.3 (R Core Team, 2020). Abbreviations of variables as in Tables 1 and 2.

Model formula	*k*	AICc	ΔAICc	W_i
**MP1~IOD_AUG_OCT_1y+NAO_APR_MAY+PSAH_AUG_OCT_1y+SCAND_JUN_JUL_1y+SOI_NOV_MAR +TSAH_AUG_OCT_1y**	**7**	**79.17**	**0.00**	**0.36**
MP1~IOD_AUG_OCT_1y+IOD_NOV_MAR+NAO_APR_MAY+NAO_NOV_MAR+SAH_AUG_OCT_1y+TSAH_NOV_MAR	7	80.60	1.43	0.18
MP1~IOD_AUG_OCT_1y+NAO_APR_MAY+NAO_NOV_MAR+PSAH_AUG_OCT_1y+SCAND_JUN_JUL_1y+SOI_NOV_MAR+ TSAH_AUG_OCT_1y	8	80.67	1.50	0.17
MP1~IOD_AUG_OCT_1y+NAO_APR_MAY+NAO_NOV_MAR+SOI_NOV_MAR+TSAH_NOV_MAR	6	80.90	1.73	0.15
MP1~IOD_AUG_OCT_1y+NAO_APR_MAY+PSAH_AUG_OCT_1y+SOI_NOV_MAR+TSAH_AUG_OCT_1y	6	81.10	1.93	0.14

Table S10. Model selection procedure by "all subsets", according to AICc, from the full model (Table S8) of the relationship between 15 climate variables and the year, and timing of migration in the second main period of spring (MP2) for Willow Warblers caught at Bukowo, Poland, in 1982–2017. The table presents all models with ΔAICc < 2. The models were ranked by corrected Akaike's Information Criteria for small samples size (AICc), *k* is the number of estimated parameters in the model, ΔAICc gives the difference in AICc from the model with lowest AICc, w_i is the Akaike weight. The best model, discussed in the text, in **bold** face. Model selection with the package MuMIn 1.43.6 (Bartoń, 2020) in R 4.0.3 (R Core Team, 2020). Abbreviations of variables as in Tables 1 and 2.

Model formula	*k*	AICc	ΔAICc	W_i
**MP2~IOD_AUG_OCT_1y+TLEB_APR_MAY+NAO_APR_MAY+PSAH_AUG_OCT_1y+SCAND_JUN_JUL_1y**	**6**	**68.09**	**0.00**	**0.18**
MP2~IOD_AUG_OCT_1y+TLEB_APR_MAY+NAO_APR_MAY+PSAH_AUG_OCT_1y+PSAH_NOV_MAR+SCAND_JUN_JUL_1y	7	68.37	0.28	0.15
MP2~IOD_AUG_OCT_1y+TLEB_APR_MAY+NAO_APR_MAY+PSAH_AUG_OCT_1y+SCAND_JUN_JUL_1y+TSAH_AUG_OCT_1y	7	68.89	0.80	0.12
MP2~IOD_AUG_OCT_1y+TLEB_APR_MAY+NAO_APR_MAY+NAO_NOV_MAR+SCAND_JUN_JUL_1y+TSAH_NOV_MAR	7	69.15	1.06	0.10
MP2~IOD_AUG_OCT_1y+TLEB_APR_MAY+NAO_APR_MAY+NAO_NOV_MAR+PSAH_AUG_OCT_1y+SCAND_JUN_JUL_1y +TSAH_NOV_MAR	8	69.18	1.09	0.10
MP2~IOD_AUG_OCT_1y+TLEB_APR_MAY+NAO_APR_MAY+NAO_NOV_MAR+SCAND_JUN_JUL_1y+SOI_AUG_OCT_1y +TSAH_NOV_MAR	8	69.27	1.18	0.10
MP2~IOD_AUG_OCT_1y+TLEB_APR_MAY+NAO_APR_MAY+NAO_NOV_MAR+SCAND_JUN_JUL_1y+SOI_NOV_MAR +TSAH_NOV_MAR	8	69.37	1.27	0.09
MP2~IOD_AUG_OCT_1y+TLEB_APR_MAY+NAO_APR_MAY+PSAH_AUG_OCT_1y+PSAH_NOV_MAR+SCAND_JUN_JUL_1y +TSAH_AUG_OCT_1y	8	69.52	1.43	0.09
MP2~IOD_AUG_OCT_1y+IOD_NOV_MAR+TLEB_APR_MAY+NAO_APR_MAY+PSAH_AUG_OCT_1y+SCAND_JUN_JUL_1y +TSAH_AUG_OCT_1y	8	69.83	1.74	0.07

Table S11. Model selection procedure by "all subsets" selection, according to AICc, from the full model (Table S8) of the relationship between 15 climate variables and the year, and timing of migration in the third main period of spring (MP3) for Willow Warblers caught at Bukowo, Poland, in 1982–2017. The table presents all models with ΔAICc < 2. The models were ranked by corrected Akaike's Information Criteria for small samples size (AICc), *k* is the number of estimated parameters in the model, ΔAICc gives the difference in AICc from the model with lowest AICc, w_i is the Akaike weight. The best model, discussed in the text, in **bold** face. Model selection conducted using package MuMIn 1.43.6 (Bartoń, 2020) in R 4.0.3 (R Core Team, 2020). Abbreviations of variables as in Tables 1 and 2.

Model formula	*k*	AICc	ΔAICc	W_i
**MP3~TLEB_APR_MAY+NAO_APR_MAY+NAO_NOV_MAR+PSAH_NOV_MAR+SCAND_JUN_JUL_1y**	**6**	**92.69**	**0.00**	**0.21**
MP3~TLEB_APR_MAY+NAO_NOV_MAR+PSAH_NOV_MAR+SCAND_JUN_JUL_1y	5	92.95	0.26	0.19
MP3~NAO_NOV_MAR+PSAH_NOV_MAR+SCAND_JUN_JUL_1y	4	93.90	1.21	0.12
MP3~NAO_NOV_MAR+PSAH_NOV_MAR	3	93.99	1.30	0.11
MP3~NAO_NOV_MAR+TSAH_AUG_OCT_1y	3	94.07	1.38	0.11
MP3~TLEB_APR_MAY+NAO_APR_MAY+PSAH_NOV_MAR+SCAND_JUN_JUL_1y	5	94.31	1.62	0.09
MP3~TLEB_APR_MAY+NAO_APR_MAY+NAO_JUN_JUL_1y+NAO_NOV_MAR+PSAH_NOV_MAR+SCAND_JUN_JUL_1y	7	94.43	1.74	0.09
MP3~NAO_APR_MAY+NAO_NOV_MAR	4	94.56	1.87	0.08

Table S12. Model selection procedure by “all subsets”, according to AICc, from the full model (Table S8) of the relationship between 15 climate variables and the year, and timing of migration for the whole spring (AA) for Willow Warblers caught at Bukowo, Poland, in 1982–2017. The table presents all models with ΔAICc < 2. The models were ranked by corrected Akaike’s Information Criteria for small samples size (AICc), *k* is the number of estimated parameters in the model, ΔAICc gives the difference in AICc from the model with lowest AICc, w_i is the Akaike weight. The best model, discussed in the text, in **bold** face. Model selection with the package MuMIn 1.43.6 (Bartoń, 2020) in R 4.0.3 (R Core Team, 2020). Abbreviations of variables as in Tables 1 and 2.

Model formula	*k*	AICc	ΔAICc	W_i
**AA~IOD_AUG_OCT_1y+TLEB_APR_MAY+NAO_APR_MAY+NAO_NOV_MAR+SCAND_JUN_JUL_1y+SOI_AUG_OCT_1y +TSAH_NOV_MAR**	**8**	**78.82**	**0.00**	**0.29**
AA~IOD_AUG_OCT_1y+TLEB_APR_MAY+NAO_APR_MAY+NAO_NOV_MAR+SCAND_JUN_JUL_1y+SOI_NOV_MAR +TSAH_NOV_MAR	8	78.82	0.00	0.29
AA~IOD_AUG_OCT_1y+TLEB_APR_MAY+NAO_APR_MAY+NAO_NOV_MAR+PSAH_NOV_MAR+SCAND_JUN_JUL_1y +SOI_AUG_OCT_1y+TSAH_NOV_MAR	9	80.25	1.43	0.14
AA~IOD_AUG_OCT_1y+TLEB_APR_MAY+NAO_APR_MAY+NAO_NOV_MAR+PSAH_NOV_MAR+SCAND_JUN_JUL_1y +SOI_AUG_OCT_1y	8	80.37	1.55	0.14
AA~IOD_AUG_OCT_1y+NAO_APR_MAY+NAO_NOV_MAR+SCAND_JUN_JUL_1y+SOI_NOV_MAR+TSAH_NOV_MAR	7	80.46	1.64	0.13

Table S13. Contributions of each explanatory variable to the top multiple regression models (ΔAICc < 2) of the relationship between 15 climate variables and the year, and the timing of migration in three main periods of spring (MP1, MP2, MP3) and over the whole season (AA) for Willow Warblers caught at Bukowo, Poland, in 1982–2017. The contribution of each variable was calculated as the sum of the Akaike weights (w_i) of the top models that include this variable (Tables S6–S9). If the variable occurs in all the top models, its contribution = 100%; if it occurs in none of the top models, its contribution = 0%. The nine climate variables selected by the best models and analysed further are marked in **bold**. SOI Aug–Oct_1y and SOI Nov–March were strongly correlated (Table S3), and never occurred in the same model (Tables S6–S9). To avoid strong multicollinearity we therefore chose only SOI Aug–Oct_1y for further analyses, because it had a greater sum of contributions in the top models for the three main periods.

No	Explanatory variable	Contributions to top models			Sum for three main periods
		MP1	MP2	MP3	
1	**NAO Apr–May**	100%	100%	48%	**248%**
2	**IOD Aug–Oct_1y**	100%	100%	0%	**200%**
3	**SCAND Jun–Jul_1y**	18%	100%	70%	**188%**
4	**NAO Nov–Mar**	50%	40%	91%	**180%**
5	**TLEB Apr–May**	0%	100%	58%	**158%**
6	**PSAH Nov–Mar**	53%	24%	81%	**158%**
7	**SAH Aug–Oct_1y**	67%	71%	0%	**138%**
8	**SOI Aug–Oct_1y**	82%	10%	0%	**92%**
9	SOI Nov_Mar	67%	9%	0%	76%
10	**TSAH Aug–Oct_1y**	33%	28%	0%	**61%**
11	TSAH Nov–Mar	0%	40%	19%	59%
12	NAO Jun–Jul_1y	0%	0%	9%	9%
13	IOD Nov–Mar	0%	7%	0%	7%
14	PSAH Aug–Oct_1y	0%	0%	0%	0%
15	SCAND Apr–May	0%	0%	0%	0%
16	YearN	0%	0%	0%	0%

Table S14. Relationships between nine overlapping sub-periods in the Annual Anomaly for the timing of Willow Warblers' spring migration at Bukowo, Poland, in 1982–2017 and nine selected climate variables. Estimate – coefficients from multiple regression; SE – standard error of the estimates; *t, p* – *t*-test and significance of each estimate, $p < 0.05$ in **bold**, $0.05 < p < 0.1$ in ***bold italics***; VIF – variance inflation factor, R^2 – partial determination coefficients, *pR* – partial correlation coefficient. Abbreviations of variables as in Tables 1 and 2.

Explanatory variable	Estimate	SE	*t*	*p*	VIF	R^2	*pR*
**SP1**	Model statistics: $F_{9,25} = 4.46$, $AdjR^2 = 47.8\%$						
TLEB_APR_MAY	–0.04	0.15	–0.25	0.801	1.48	0.00	–0.05
NAO_APR_MAY	–0.25	0.13	–1.90	***0.069***	1.14	0.13	***–0.36***
NAO_NOV_MAR	–0.26	0.14	–1.90	***0.069***	1.25	0.13	***–0.36***
PSAH_NOV_MAR	–0.13	0.13	–1.02	0.317	1.10	0.04	–0.20
**SOI_AUG_OCT_1y**	–0.62	0.17	–3.60	**0.001**	1.95	0.34	**–0.58**
**IOD_AUG_OCT_1y**	–0.56	0.18	–3.07	**0.005**	2.18	0.27	**–0.52**
PSAH_AUG_OCT_1y	–0.29	0.16	–1.84	***0.077***	1.58	0.12	***–0.35***
TSAH_AUG_OCT_1y	–0.30	0.15	–2.01	***0.055***	1.44	0.14	***–0.37***
SCAND_JUN_JUL_1y	–0.19	0.15	–1.26	0.220	1.46	0.06	–0.24
**SP2**	Model statistics: $F_{9,25} = 4.85$, $AdjR^2 = 50.5\%$						
TLEB_APR_MAY	–0.04	0.15	–0.25	0.804	1.48	0.00	–0.05
**NAO_APR_MAY**	–0.28	0.13	–2.20	**0.037**	1.14	0.16	**–0.40**
NAO_NOV_MAR	–0.23	0.13	–1.73	***0.095***	1.25	0.11	***–0.33***
PSAH_NOV_MAR	–0.11	0.13	–0.86	0.401	1.10	0.03	–0.17
**SOI_AUG_OCT_1y**	–0.51	0.17	–3.00	**0.006**	1.95	0.26	**–0.51**
**IOD_AUG_OCT_1y**	–0.51	0.18	–2.88	**0.008**	2.18	0.25	**–0.50**
**PSAH_AUG_OCT_1y**	–0.37	0.15	–2.41	**0.023**	1.58	0.19	**–0.43**
**TSAH_AUG_OCT_1y**	–0.32	0.14	–2.21	**0.037**	1.44	0.16	**–0.40**
SCAND_JUN_JUL_1y	–0.25	0.15	–1.71	0.100	1.46	0.10	–0.32
**SP3**	Model statistics: $F_{9,25} = 8.67$, $AdjR^2 = 67.0\%$						
**TLEB_APR_MAY**	–0.28	0.12	–2.36	**0.026**	1.48	0.18	**–0.43**
**NAO_APR_MAY**	–0.40	0.11	–3.75	**0.001**	1.14	0.36	**–0.60**
NAO_NOV_MAR	–0.14	0.11	–1.27	0.215	1.25	0.06	–0.25
PSAH_NOV_MAR	–0.10	0.10	–0.94	0.358	1.10	0.03	–0.18
**SOI_AUG_OCT_1y**	–0.29	0.14	–2.12	**0.044**	1.95	0.15	**–0.39**
**IOD_AUG_OCT_1y**	–0.40	0.15	–2.78	**0.010**	2.18	0.24	**–0.49**
**PSAH_AUG_OCT_1y**	–0.38	0.12	–3.04	**0.005**	1.58	0.27	**–0.52**
**TSAH_AUG_OCT_1y**	–0.29	0.12	–2.50	**0.019**	1.44	0.20	**–0.45**
**SCAND_JUN_JUL_1y**	–0.50	0.12	–4.20	**0.000**	1.46	0.41	**–0.64**
**SP4**	Model statistics: $F_{9,25} = 10.13$, $AdjR^2 = 70.7\%$						
**TLEB_APR_MAY**	–0.41	0.11	–3.59	**0.001**	1.48	0.34	**–0.58**
**NAO_APR_MAY**	–0.47	0.10	–4.72	**0.000**	1.14	0.47	**–0.69**
NAO_NOV_MAR	–0.13	0.10	–1.22	0.232	1.25	0.06	–0.24
PSAH_NOV_MAR	–0.10	0.10	–1.07	0.294	1.10	0.04	–0.21
SOI_AUG_OCT_1y	–0.19	0.13	–1.45	0.160	1.95	0.08	–0.28
**IOD_AUG_OCT_1y**	–0.37	0.14	–2.74	**0.011**	2.18	0.23	**–0.48**
**PSAH_AUG_OCT_1y**	–0.31	0.12	–2.66	**0.014**	1.58	0.22	**–0.47**
**TSAH_AUG_OCT_1y**	–0.22	0.11	–2.01	**0.056**	1.44	0.14	**–0.37**
**SCAND_JUN_JUL_1y**	–0.52	0.11	–4.64	**0.000**	1.46	0.46	**–0.68**

Table S14. continued

Explanatory variable	Estimate	SE	*T*	*p*	VIF	R^2	*pR*
**SP5** Model statistics: $F_{9,25}$ = 8.35, AdjR^2 = 66.0%							
**TLEB_APR_MAY**	–0.44	0.12	–3.60	**0.001**	1.48	0.34	**–0.58**
**NAO_APR_MAY**	–0.48	0.11	–4.46	**0.000**	1.14	0.44	**–0.67**
NAO_NOV_MAR	–0.14	0.11	–1.24	0.228	1.25	0.06	–0.24
PSAH_NOV_MAR	–0.15	0.10	–1.46	0.157	1.10	0.08	–0.28
SOI_AUG_OCT_1y	–0.16	0.14	–1.14	0.266	1.95	0.05	–0.22
**IOD_AUG_OCT_1y**	–0.40	0.15	–2.71	**0.012**	2.18	0.23	**–0.48**
PSAH_AUG_OCT_1y	–0.23	0.13	–1.82	***0.081***	1.58	0.12	***–0.34***
TSAH_AUG_OCT_1y	–0.16	0.12	–1.30	0.205	1.44	0.06	–0.25
**SCAND_JUN_JUL_1y**	–0.48	0.12	–4.00	**0.001**	1.46	0.39	**–0.62**
**SP6** Model statistics: $F_{9,25}$= 6.32, AdjR^2 = 58.5%							
**TLEB_APR_MAY**	–0.43	0.13	–3.21	**0.004**	1.48	0.29	**–0.54**
**NAO_APR_MAY**	–0.47	0.12	–3.99	**0.001**	1.14	0.39	**–0.62**
NAO_NOV_MAR	–0.19	0.12	–1.53	0.139	1.25	0.09	–0.29
**PSAH_NOV_MAR**	–0.26	0.12	–2.22	**0.035**	1.10	0.17	**–0.41**
SOI_AUG_OCT_1y	–0.13	0.15	–0.85	0.403	1.95	0.03	–0.17
**IOD_AUG_OCT_1y**	–0.41	0.16	–2.50	**0.019**	2.18	0.20	**–0.45**
PSAH_AUG_OCT_1y	–0.07	0.14	–0.54	0.594	1.58	0.01	–0.11
TSAH_AUG_OCT_1y	–0.10	0.13	–0.75	0.460	1.44	0.02	–0.15
**SCAND_JUN_JUL_1y**	–0.46	0.13	–3.46	**0.002**	1.46	0.32	**–0.57**
**SP7** Model statistics: $F_{9,25}$= 3.21, AdjR^2 =36.9%							
**TLEB_APR_MAY**	–0.41	0.17	–2.46	**0.021**	1.48	0.19	**–0.44**
**NAO_APR_MAY**	–0.38	0.15	–2.60	**0.015**	1.14	0.21	**–0.46**
NAO_NOV_MAR	–0.28	0.15	–1.81	***0.082***	1.25	0.12	***–0.34***
**PSAH_NOV_MAR**	–0.30	0.14	–2.13	**0.043**	1.10	0.15	**–0.39**
SOI_AUG_OCT_1y	–0.02	0.19	–0.11	0.915	1.95	0.00	–0.02
IOD_AUG_OCT_1y	–0.24	0.20	–1.17	0.252	2.18	0.05	–0.23
PSAH_AUG_OCT_1y	0.06	0.17	0.33	0.743	1.58	0.00	0.07
TSAH_AUG_OCT_1y	–0.01	0.16	–0.06	0.954	1.44	0.00	–0.01
**SCAND_JUN_JUL_1y**	–0.41	0.16	–2.51	**0.019**	1.46	0.20	**–0.45**
**SP8** Model statistics: $F_{9,25}$= 1.84, AdjR^2 = 18.3%							
TLEB_APR_MAY	–0.30	0.19	–1.61	0.120	1.48	0.09	–0.31
NAO_APR_MAY	–0.28	0.17	–1.70	0.102	1.14	0.10	–0.32
NAO_NOV_MAR	–0.29	0.17	–1.67	0.108	1.25	0.10	–0.32
PSAH_NOV_MAR	–0.33	0.16	–2.03	***0.053***	1.10	0.14	***–0.38***
SOI_AUG_OCT_1y	0.01	0.22	0.05	0.960	1.95	0.00	0.01
IOD_AUG_OCT_1y	–0.13	0.23	–0.55	0.586	2.18	0.01	–0.11
PSAH_AUG_OCT_1y	0.04	0.20	0.21	0.836	1.58	0.00	0.04
TSAH_AUG_OCT_1y	–0.05	0.19	–0.27	0.792	1.44	0.00	–0.05
SCAND_JUN_JUL_1y	–0.35	0.19	–1.87	***0.074***	1.46	0.12	***–0.35***
**SP9** Model statistics: $F_{9,25}$= 1.28, AdjR^2 = 0.7%							
TLEB_APR_MAY	–0.15	0.20	–0.75	0.462	1.48	0.02	–0.15
NAO_APR_MAY	–0.16	0.18	–0.89	0.381	1.14	0.03	–0.18
NAO_NOV_MAR	–0.22	0.18	–1.20	0.241	1.25	0.05	–0.23
**PSAH_NOV_MAR**	–0.41	0.17	–2.36	**0.027**	1.10	0.18	–0.43
SOI_AUG_OCT_1y	0.01	0.23	0.06	0.953	1.95	0.00	0.01
IOD_AUG_OCT_1y	–0.09	0.24	–0.37	0.717	2.18	0.01	–0.07
PSAH_AUG_OCT_1y	–0.06	0.21	–0.26	0.793	1.58	0.00	–0.05
TSAH_AUG_OCT_1y	–0.05	0.20	–0.27	0.788	1.44	0.00	–0.05
SCAND_JUN_JUL_1y	–0.29	0.20	–1.46	0.158	1.46	0.08	–0.28

Table S15. Partial correlation coefficients for each of nine selected climate variables (Table 1) in multiple regression models with the response variable as each of nine overlapping sub-periods of the Annual Anomaly (AA) for the timing of Willow Warblers' spring migration at Bukowo, Poland, in 1982–2017. The partial correlation coefficients are the same as in the last column of Table S14, compiled for the sub-periods of AA. This is the source data for Fig. 4A–I, but in those figures the signs of the correlations were inverted to better visualise the change in relationships with the progress of passage.

**Symbols of sub-periods**	**SP1**	**SP2**	**SP3**	**SP4**	**SP5**	**SP6**	**SP7**	**SP8**	**SP9**
Percentiles at multiyear curve for sub-periods	0–20%	11–30%	21–40%	31–50%	41–60%	51–70%	61–80%	71–90%	81–100%
Dates of sub-periods/ Climate variable	1–22 Apr	18–26 Apr	23–28 Apr	26Apr– 1 May	29Apr– 3 May	2–5 May	4–8 May	6–11 May	9–15 May
TLEB_APR_MAY	–0.05	–0.05	–0.43	–0.58	–0.58	–0.54	–0.44	–0.31	–0.15
NAO_APR_MAY	–0.36	–0.40	–0.60	–0.69	–0.67	–0.62	–0.46	–0.32	–0.18
NAO_NOV_MAR	–0.36	–0.33	–0.25	–0.24	–0.24	–0.29	–0.34	–0.32	–0.23
PSAH_NOV_MAR	–0.20	–0.17	–0.18	–0.21	–0.28	–0.41	–0.39	–0.38	–0.43
SOI_AUG_OCT_1y	–0.58	–0.51	–0.39	–0.28	–0.22	–0.17	–0.02	0.01	0.01
IOD_AUG_OCT_1y	–0.52	–0.50	–0.49	–0.48	–0.48	–0.45	–0.23	–0.11	–0.07
PSAH_AUG_OCT_1y	–0.35	–0.43	–0.52	–0.47	–0.34	–0.11	0.07	0.04	–0.05
TSAH_ AUG_OCT_1y	–0.37	–0.40	–0.45	–0.37	–0.25	–0.15	–0.01	–0.05	–0.05
SCAND_JUN_JUL_1y	–0.24	–0.32	–0.64	–0.68	–0.62	–0.57	–0.45	–0.35	–0.28

Table S16. Summary statistics for linear regressions of Annual Anomaly in spring migration timing for three main periods and for the whole spring season with the count of juvenile Willow Warblers caught the previous autumn (Njuv_1y) at Bukowo, Poland, in 1982–2017. The exceptionally high Njuv in autumn 1982 was excluded from this analysis as an outlier. *ß* slope = regression coefficient; SE = Standard Error; R^2 – determination coefficient; *t, p* = results of two-sided *t*-test, significant regressions after Benjamini-Hochberg correction for multiple comparisons marked in **bold** face. These regressions are plotted in Fig. 5.

Dependent variable	*ß* slope	SE	R^2	t_{34}	*p*
MP1	**0.45**	0.16	0.21	2.85	**0.008**
MP2	**0.55**	0.15	0.31	3.66	**0.001**
MP3	0.34	0.17	0.12	1.97	0.058
AA	**0.53**	0.15	0.29	3.47	**0.002**

REFERENCES

Bartoń K. 2019. Package 'MuMIn'. Multi-Model Inference. *Available at https://cran.r-project.org/web/packages/MuMIn/MuMIn.pdf.* (accessed November 20, 2019).

Benjamini Y., Hochberg Y. 1995. Controlling the false discovery rate: a practical and powerful approach to multiple testing. *Journal of the Royal Statistical Society, Series B* 57 (1): 289–300.

Crawley MJ. 2013. *The R Book*. Chichester. England: John Wiley & Sons.

R Core Team. 2020. R: a language and environment for statistical computing. Available at https://www.r-project.org (accessed February 5, 2021).
